# Epithelial to Mesenchymal Transition Is Mechanistically Linked with Stem Cell Signatures in Prostate Cancer Cells

**DOI:** 10.1371/journal.pone.0012445

**Published:** 2010-08-27

**Authors:** Dejuan Kong, Sanjeev Banerjee, Aamir Ahmad, Yiwei Li, Zhiwei Wang, Seema Sethi, Fazlul H. Sarkar

**Affiliations:** Department of Pathology, Karmanos Cancer Institute, Wayne State University School of Medicine, Detroit, Michigan, United States of America; Baylor College of Medicine, United States of America

## Abstract

**Background:**

Current management of patients diagnosed with prostate cancer (PCa) is very effective; however, tumor recurrence with Castrate Resistant Prostate Cancer (CRPC) and subsequent metastasis lead to poor survival outcome, suggesting that there is a dire need for novel mechanistic understanding of tumor recurrence, which would be critical for designing novel therapies. The recurrence and the metastasis of PCa are tightly linked with the biology of prostate cancer stem cells or cancer-initiating cells that is reminiscent of the acquisition of Epithelial to Mesenchymal Transition (EMT) phenotype. Increasing evidence suggests that EMT-type cells share many biological characteristics with cancer stem-like cells.

**Methodology/Principal Findings:**

In this study, we found that PCa cells with EMT phenotype displayed stem-like cell features characterized by increased expression of Sox2, Nanog, Oct4, Lin28B and/or Notch1, consistent with enhanced clonogenic and sphere (prostasphere)-forming ability and tumorigenecity in mice, which was associated with decreased expression of miR-200 and/or let-7 family. Reversal of EMT by re-expression of miR-200 inhibited prostasphere-forming ability of EMT-type cells and reduced the expression of Notch1 and Lin28B. Down-regulation of Lin28B increased let-7 expression, which was consistent with repressed self-renewal capability.

**Conclusions/Significance:**

These results suggest that miR-200 played a pivotal role in linking the characteristics of cancer stem-like cells with EMT-like cell signatures in PCa. Selective elimination of cancer stem-like cells by reversing the EMT phenotype to Mesenchymal-Epithelial Transition (MET) phenotype using novel agents would be useful for the prevention of tumor recurrence especially by eliminating those cells that are the “Root Cause” of tumor development and recurrence.

## Introduction

Emerging evidence suggest that most solid tumors including prostate cancer (PCa) may arise from cancer stem cells [1]. The cancer stem cells are cells within a tumor that possess the capacity of self-renewal and tumor-initiating capacity, and differentiate into the heterogeneous lineages of cancer cells that comprise in a tumor mass. These tumor-initiating cells could provide a reservoir for cells that cause tumor recurrence after therapy. Wang et al. have shown that the origin of PCa cells is from the differentiated luminal epithelial stem cells [2]. However, there is a greater degree of phenotypic heterogeneity of cells in PCa, especially within metastatic sites. Metastases often include rare cells that are phenotypically undifferentiated [3], suggesting that metastases-initiating cells may not be the only cells that are derived from androgen receptor-positive luminal cells. Although origin of PCa cells needs to be fully elucidated, a number of mounting evidence suggest that tumor-initiating cells or cancer stem cells play a critical role in the progression and recurrence of PCa to castrate resistant prostate cancer (CRPC) and its subsequent metastasis.

Recent studies have indicated that somatic cells can be reprogrammed into pluripotent embryonic stem-like cells by co-expression of pluripotency stem cell markers such as Oct4, Sox2, Nanog and Lin28 [4], [5], which raises the possibility that combined expression of stem cell-associated factors and special oncogenes could also induce a undifferentiated state in cancer cells. Interestingly, cell lines derived from human PCa specimen showed epithelial phenotype. However, immortalization of these cells by hTERT showed the expression of pluripotency stem cell markers, including Oct4, Nonag, and Sox2 [6], which could be associated with disease progression because over-expression of Oct4, Sox2, Nanog and c-myc has been found in poorly differentiated tumors [7]. Jeter et al. have shown that knock-down of Nanog in PCa cells significantly decreased long-term clonogenic growth and inhibited tumor growth [8]. Oct4 has also been shown to play important roles in the progression of PCa [9].

Epithelial to mesenchymal transition (EMT) is a vital process for morphogenesis during embryonic development, but more recently it has also been implicated in the conversion of early stage tumors into invasive malignancies [10]. Progression of most carcinomas toward malignancy is associated with the loss of epithelial differentiation and by switching toward mesenchymal phenotype, which is accompanied by increased cell motility and invasion. Recent studies have demonstrated that EMT plays a critical role not only in tumor metastasis but also in tumor recurrence that is believed to be tightly linked with the biology of cancer stem-like cells or cancer-initiating cells [11]–[13]. However, the mechanisms by which EMT cells generate the stem-like cells remain to be elucidated. MicroRNAs (miRNAs) are emerging as master regulators of cell differentiation and involved in the acquisition of EMT phenotype during tumor progression [14]. Two evolutionary conserved families, miR-200 and let-7 have been shown to regulate the differentiation processes during the development. Interestingly, recent studies have also shown that miR-200 family not only could regulate the processes of EMT by targeting E-box binding protein ZEB1 and ZEB2 [15] but was also associated with stem-like cell signatures by regulating the expression of Bmi1 [16], [17]. Let-7 family of miRNAs has been shown to act as a tumor suppressor through targeting Ras, high mobility group A2 (HMGA2) and c-myc, and decreased let-7 expression has been linked with increased tumorigenicity and poor patient prognosis. More importantly, Let-7 family members regulate the self-renewal of breast cancer cells [18], which is associated with stem cell phenotype. Recent studies have also documented that lin28B could block the accumulation of mature let-7 [19], which in turn regulates “stemness” by inhibiting self-renewal, the cellular characteristics of cancer stem-like cells associated with tumor recurrence. The recurrence of PCa is believed to be strongly linked with the biology of prostate cancer stem cells or cancer-initiating cells [11], [20], [21], while increasing evidence have shown that cells with EMT induced by different factors are rich source of cancer stem-like cells [12], [13], [22], [23].

Therefore, it is important to identify which factors could induce EMT and how the EMT cells could become a resource for cancer stem-like cells, which further underscores the mechanistic role of such factors toward the development of novel and targeted therapies for CRPC. Studies have shown that platelet derived growth factor (PDGF) signaling contributes to EMT [24], [25]. Recently, we and others have shown that PDGF-D could regulate cancer cell invasion and angiogenesis, which was consistent with the acquisition of EMT phenotype [26]–[30]. Interestingly, increased expression of PDGF-D has also been found in human PCa [31], suggesting that PDGF-D could play important roles in the progression of human PCa contributed by EMT-phenotypic or cancer stem-like cells. In this study, we found that PDGF-D over-expressing PC3 cells acquired EMT phenotype and shared stem-like cell features characterized by increased expression of stem cell markers such as Notch1, Sox2, Nanog, Oct4 and Lin28B, which was consistent with enhanced clonogenic, prostasphere-forming ability as well as increased tumorigenicity in mice. Concomitantly, we found ARCaP_M_ cells with EMT phenotype also shared stem-like cell signatures characterized by increased expression of transcription factor Notch1 and enhanced clonogenic, prostasphere-forming ability compared with control cells (ARCaP_E_ cells) with epithelial phenotype. These EMT-type cells also showed decreased expression of miR-200 or let-7, and reversal of EMT by forcing expression of miR-200 significantly inhibited clonogenic and prostasphere-forming ability, which was consistent with the inhibition of Notch1 and Lin28B expression. Moreover, knock-down of Lin28B markedly increased let-7 expression, suggesting that miR-200 and let-7 could act as a target for the prevention of tumor recurrence and metastasis in PCa.

## Results

### Clonogenic ability was increased in PC3 PDGF-D and ARCaP_M_ cells having EMT phenotype

**Figure 1:**
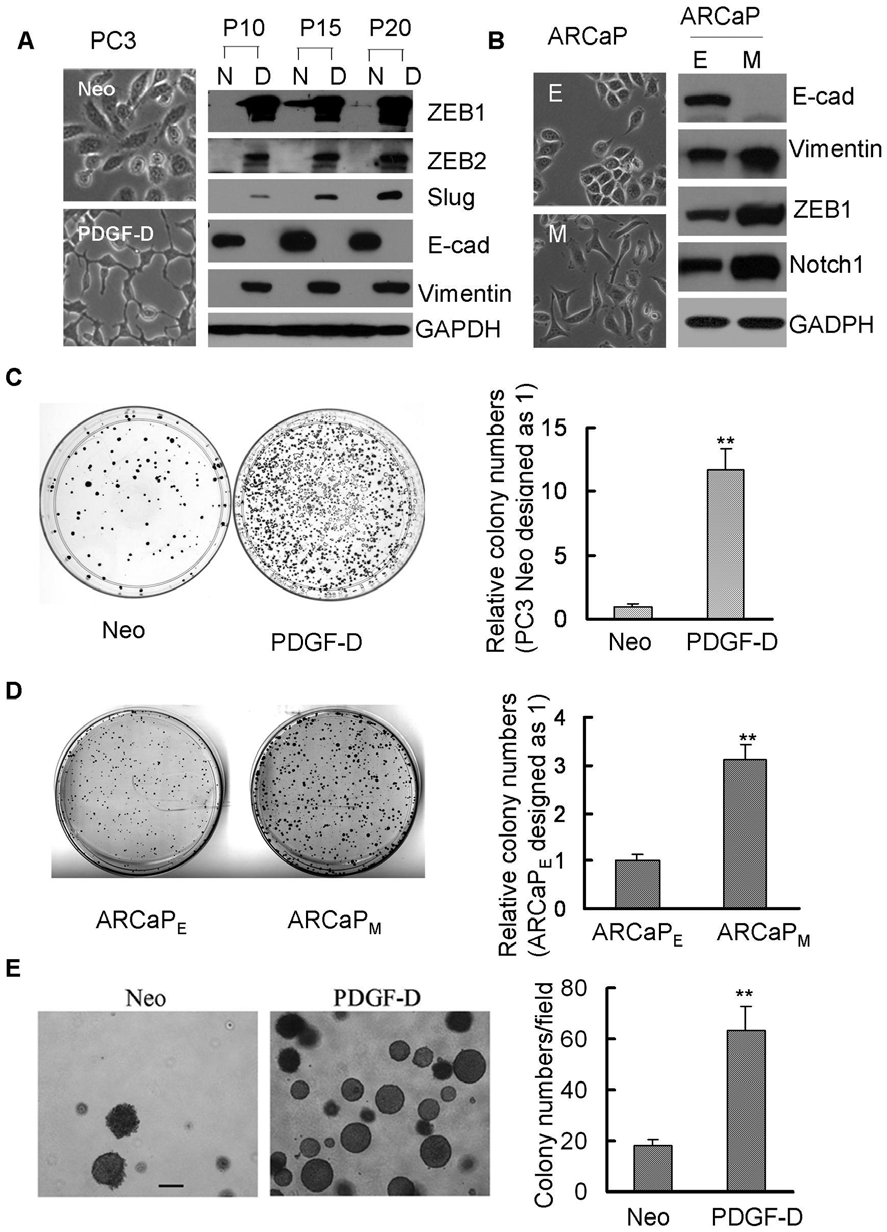
Cells with EMT signatures displayed increased Clonogenic ability. (A) Photographs of cells were shown: PC3 Neo cells displayed rounded epithelial cell shape and PC3 PDGF-D cells exhibited a fibroblastic-type phenotype (left panel, original magnification, 200 X). Western blot analysis showed the expression of transcription repressors associated with EMT, and mesenchymal as well as epithelial markers in PC3 Neo and PC3 PDGF-D cells (right panel, passage 10 to 20). (B) The morphology of ARCaP_M_ and ARCaP_E_ cells was shown (left panel).Western blot analysis indicated the increased ZEB1, vimentin and Notch1 expression and decreased E-cadherin expression in ARCaP_M_ cells compared with ARCaP_E_ (right panel). (C) and (D) Photographs of colonies from PC3 Neo and PC3 PDGF-D or ARCaP_M_ and ARCaP_E_ were shown (left panel). The colony numbers were counted and the data was presented as relative colony numbers of PC3 Neo or ARCaP_E_ designed as 1 (right panel). (E) The colonies grown on soft agar were photographed (left panel, bar, 200 µm). The colony numbers were counted under a phase contrast microscope. Data was presented as colony numbers per field (right panel). **, p<0.01 compared to Neo or ARCaP_E_ cells (N: PC3 Neo cells, D: PC3 PDGF-D cells,p10: passage 10, E-cad: E-cadherin).

The results from Western blot analysis and cell morphology as well as our published data have demonstrated that over-expression of PDGF-D induced EMT phenotype in PC3 cells [26], [28] (Fig. 1A), which was consistent with higher levels of PDGF-D in cell lysates and conditioned medium (CM) from PC3 PDGF-D cells compared with PC3 Neo cells (Fig. S1A and S1B). ARCaP_M_ cells having mesenchymal morphology showed an increased expression of mesenchymal markers and decreased expression of epithelial markers compared to ARCaP_E_ cells with epithelial phenotype (Fig. 1B). To determine whether cells with EMT phenotype could have stem-like cell characteristics, we performed clonogenic assay. We found that clonogenic ability was significantly increased in PC3 PDGF-D cells compared with PC3 Neo cells (Fig. 1C, left panel). Fig. 1C, right panel, further indicated that PC3 PDGF-D cells showed an 11-fold increase in colony numbers relative to PC3 Neo cells. ARCaP_M_ cells also displayed an increased clonogenic capacity showing 3-fold increase in colony numbers compared to ARCaP_E_ cells (Fig. 1D). Soft agar assay, also known as anchorage independent growth assay, was performed. PC3 PDGF-D cells showed dramatic increase in clonogenic potential compared to PC3 Neo cells (Fig. 1E, left panel). Fig. 1E, right panel, clearly showed the colony numbers generated from PC3 Neo and PC3 PDGF-D cells per microscopic field (40 X). These results demonstrated an increase in clonogenic ability of EMT cells.

### Self-renewal capacity was increased by EMT cells

**Figure 2:**
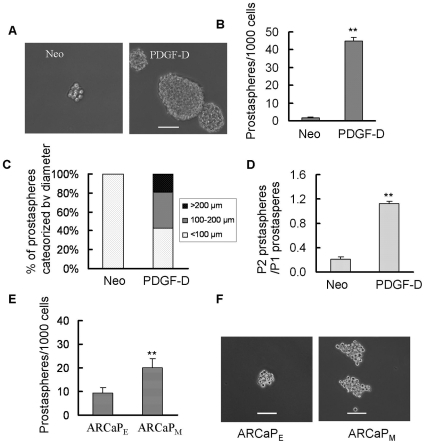
Prostaspheres were increased in cells having EMT phenotype. (A) Prostaspheres from PC3 Neo and PC3 PDGF-D cells were photographed and shown (bar, 100 µm). (B) The numbers of prostaspheres were counted under microscope. (C) Prostaspheres were photographed and the size of prostaspheres in diameter was measured using software image analysis program Scion Image. (D) The prostaspheres (P1) were collected and re-plated at a density of one cell per well in 96-well plate with ultra low attachment. After 12 days of incubation, the numbers of prostaspheres (P2) were counted under a phase contrast microscope. (E) The numbers of prostaspheres from ARCaP_E_ and ARCaP_M_ cells were counted under microscope. (F) Prostaspheres from ARCaP_E_ and ARCaP_M_ cells were photographed and shown (bar, 100 µm). **, p<0.01 compared to Neo or ARCaP_E_ cells (Neo: PC3 Neo cells, PDGF-D: PC3 PDGF-D cells).

To further determine whether cells with EMT phenotype could show stem-like cell characteristics, we tested sphere-forming (termed as prostasphere) ability of PC3 PDGF-D cells compared to PC3 Neo as well as ARCaP_M_ cells compared to ARCaP_E_ cells when grown in suspension cultures, which is an *in vitro* measure of stem-like cell characteristics. We found that PC3 PDGF-D cells showed significantly increased ability to form prostaspheres relative to PC3 Neo cells (Fig. 2A and 2B). The prostaspheres from PC3 Neo were less than 100 µm in diameter after 6 days, while 40% of the prostaspheres from PC3 PDGF-D cells were 100–200 µm, 20% of the prostaspheres from PC3 PDGF-D cells were greater than 200 µm (Fig. 2C). In order to further confirm whether the prostaspheres are the progeny of individual cells rather than the aggregates of multiple cells, the prostaspheres (P1) were collected and the cells were plated at one cell per well in 96-well plate with ultra low attachment. After 12 days, we found that one to two new prostaspheres were generated from single PC3 PDGF-D cells, while only 20% single cells of PC3 Neo generated new prostaspheres (P2, Fig. 2D). ARCaP_M_ cells also exhibited an increased prostasphere-forming capacity (Fig. 2E and 2F), suggesting that cells with EMT phenotype have acquired increased self-renewal capacity. Based on these results, further mechanistic experiments were focused using PC3 PDGF-D cells compared to PC3 Neo cells and compared with ARCaP_M_ and ARCaP_E_ cells wherever warranted.

### Gene expression profiling of stem cell markers in cells with EMT phenotype

**Figure 3:**
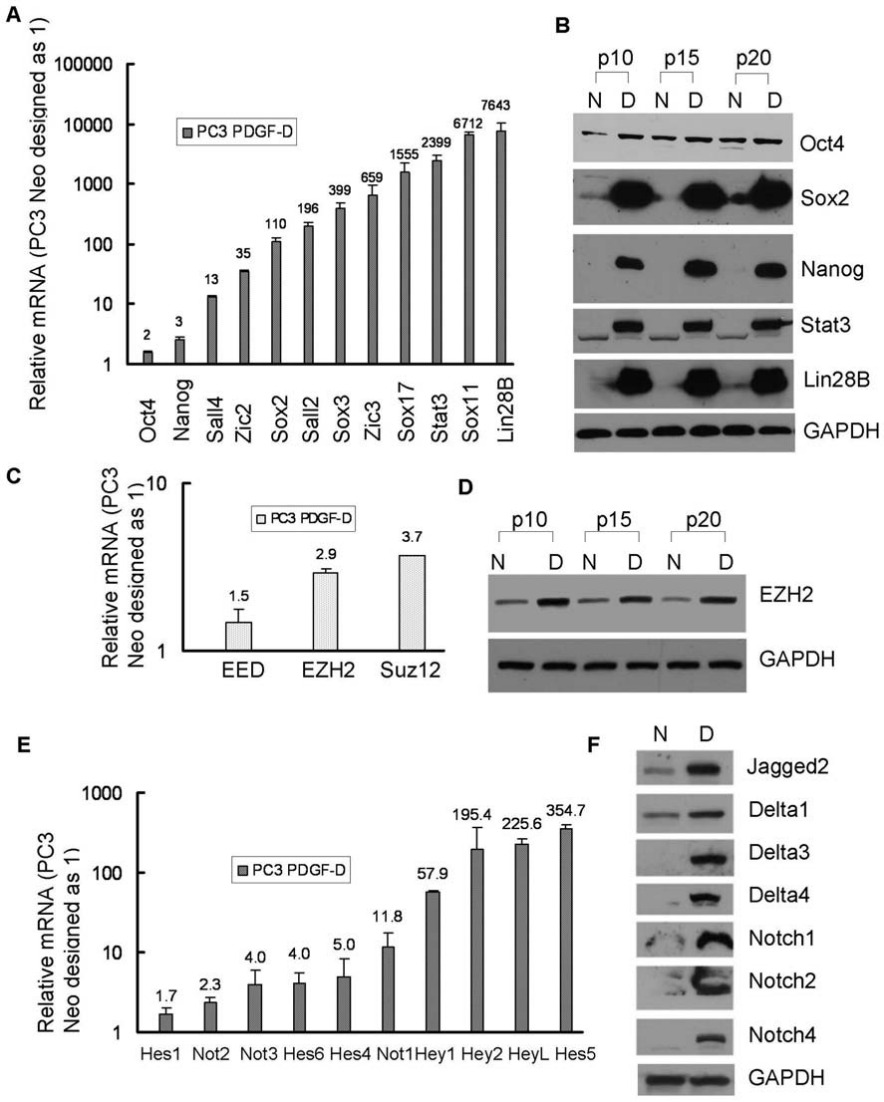
Gene expression profiling of stem cell markers in PC3 PDGF-D cells with EMT signatures. (A) The expression of genes at the mRNA levels for Oct4, Nanog, Sox family genes and Lin28B in PC3 Neo and PC3 PDGF-D cells were determined by using real time RT-PCR. (B) The results from Western blot showed that the expressions of Oct4 Sox2, Nanog, Stat3 and Lin28B were significantly increased in PC3 PDGF-D cells. (C) Real time RT-PCR was used to quantify the mRNA expression of EED, EZH2 and Suz12a. Relative mRNA levels were normalized to GAPDH. (D) The results from Western blot showed that the expression of EZH2 was significantly increased in PC3 PDGF-D cells. (E) The mRNA levels of Notch signaling factors in PC3 Neo and PC3 PDGF-D cells were determined by using real time RT-PCR. (F) The results from Western blot showed that the expressions of Notch and Notch ligands were significantly increased in PC3 PDGF-D cells. GAPDH was used for protein loading control. (N: PC3 Neo cells, D: PC3 PDGF-D cells,p10: passage 10).

To discover the genes regulating and maintaining the stem cell phenotype of PC3 PDGF-D cells having EMT signatures, we performed microarray to determine gene expression profiling of PC3 PDGF-D cells compared to PC3 Neo cells. We found that PC3 PDGF-D cells showed dramatic changes in expression of genes associated with EMT phenotype (Table S1). Interestingly, transcription factors Sox2, Nanog, Oct4 and Lin28B, known to be sufficient to reprogram mouse or human somatic cells to undifferentiated, pluripotent stem cells, were found to be significantly increased in PC3 PDGF-D cells compared to PC3 Neo cells. Concomitantly, downstream targets of these transcription factors such as Zic2, Zic3, Sall2 and Sall4 were also significantly up-regulated (Table S2). To further confirm the results from microarray gene expression analyses, we have conducted real time RT-PCR and Western blot analysis of selected genes. The results from real time RT-PCR showed 2 to thousand-fold increase in mRNA levels of these transcription factors and their downstream targets in PC3 PDGF-D cells (Fig. 3A). Western blot analysis also demonstrated that the protein expressions of Sox2, Nanog, Stat3, Lin28B and Oct4 were significantly higher in PC3 PDGF-D cells compared with PC3 Neo cells from passage 10 to passage 20 (Fig. 3B).

Polycomb group proteins are known to be involved in the regulation of gene repression through chromatin modifications, which is essential for the maintenance of the embryonic and adult stem cells [32], [33]. Polycomb repressive complex 2 (PRC2) contains Suz12, EZH2, EED and RbAp subunits and functions in the embryonic and adult stem cells to repress developmental genes that are preferentially activated during differentiation. Moreover, further studies have shown that PRC2 target genes are co-occupied by stem cell regulators such as Oct4, Sox2 and Nanog [34]. During the data analysis of our microarray data, we found that the levels of EZH2 and Suz12a mRNA were increased in PC3 PDGF-D cells compared to PC3 Neo cells (Table S2), which were further confirmed by real time RT-PCR (Fig. 3C). In addition, the protein levels of EZH2 was significantly up-regulated in PC3 PDGF-D cells relative to PC3 Neo cells (Fig. 3D) and these results clearly suggest that the EMT-type characteristics of PC3 PDGF-D cells are consistent with the signatures of stem cells or cancer stem-like cells.

Notch signaling has been shown to play important roles in pluripotency and self-renewal capacity of both embryonic and adult stem cells [35], [36]. The results from our microarray data showed that mRNA levels of Notch, Notch ligands, delta-like, Jagged as well as Notch downstream targets such as Hes and Hey were significantly higher in PC3 PDGF-D cells compared to PC3 Neo cells (Table S2). Real time RT-PCR showed 2 to 350-fold increase in mRNA levels of Notch signaling genes (Fig. 3E), which were further confirmed by Western blot analysis as shown in Fig. 3F. Likewise, we also found significantly increased level of Notch1 expression in ARCaP_M_ cells compared to ARCaP_E_ cells (Fig. 1B, right panel).

### MiR-200b and miR-200c expression linked cancer stem cell signatures with EMT phenotype

**Figure 4:**
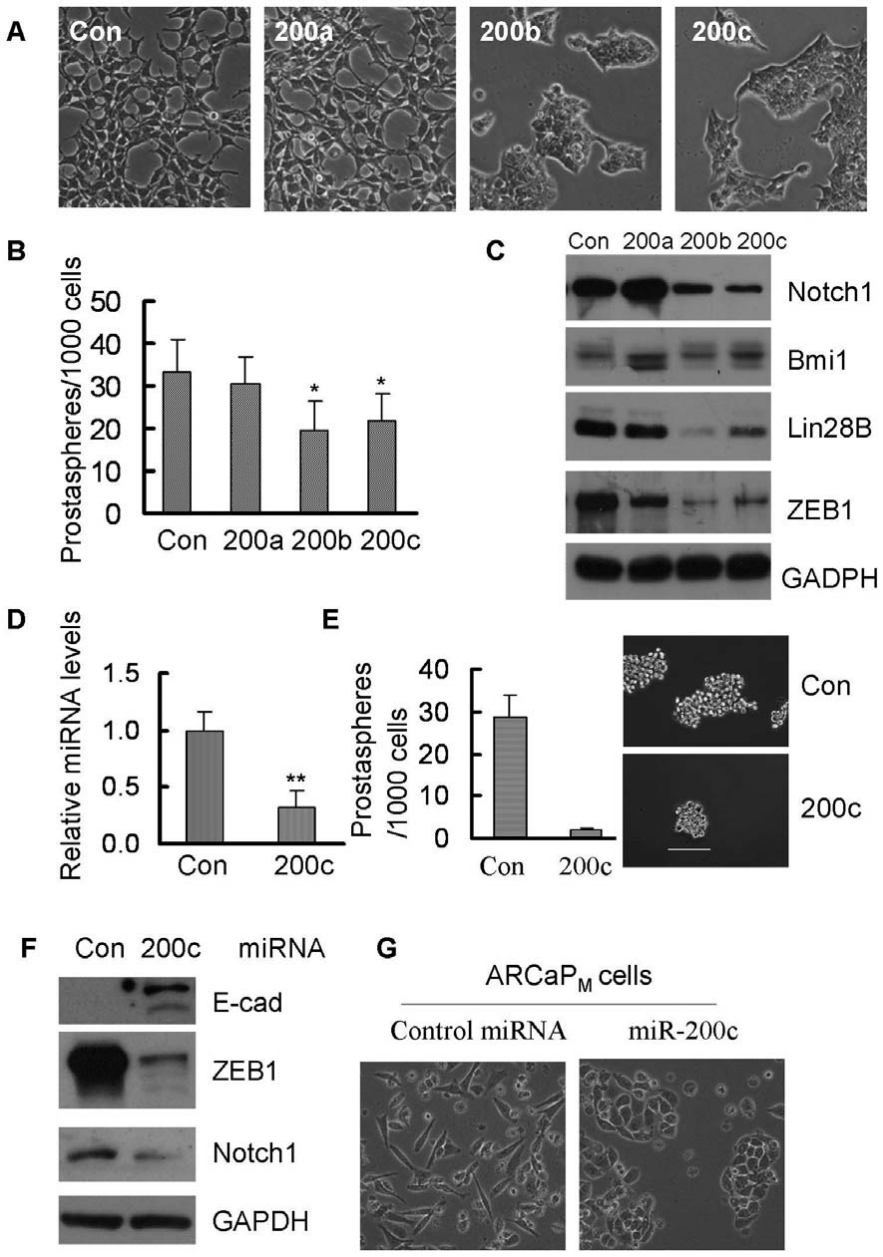
MiR-200 repressed the self-renewal capacity by regulating Notch1 and Lin28B expression. PC3 PDGF-D cells were transfected with pre-miR-200. 3 days after transfection, cells were split and transfected repeatedly with pre-miR-200 every 3–4 days for 14 days. (A) Photographs of cells are shown: transfection of PC3 PDGF-D cells with pre-miR-200 for 14 days, miR-200b and miR-200c reversed EMT cells to MET cell morphology. (B) The cells were collected for generation of prostaspheres after 14-day transfection with pre-miR-200. MiR-200b and miR-200c reduced the number of prostaspheres. (C) Western Blot showing that Notch1, Lin28B and ZEB1 expressions were down-regulated in PC3 PDGF-D cells transfected with miR-200b and miR-200c. (D) The level of miR-200c significantly decreased in ARCaP_M_ by using real time RT-PCR. (E) Transfection of pre-miR-200c after 6 days reduced the prostasphere-forming ability in ARCaP_M_ cells (Bar:100 µm). (F) Western blot showed that re-expression of miR-200c by transfection of pre-miR-200c increased the E-cadherin expression and repressed expression of ZEB1 and Notch1 in ARCaP_M_ cells, which consistent with the change from mesenchymal to epithelial cell morphology (G). *, p<0.05 compared to control; **, p<0.01 compared to control. (Con: control, 200a: pre-miR-200a, 200b: pre-miR-200b, 200c: pre-miR-200c, E-cad: E-cadherin).

In our previous studies, we found significant down-regulation of miR-200 family in PC3 PDGF-D cells with EMT phenotype [28], which was consistent with our miRNA microarray data (Table S3). To reveal whether expressions of miR-200 family are associated with stem cell signatures, PC3 PDGF-D cells were transfected with miR-200a, miR-200b and miR-200c. We found that transfection of miR-200b and miR-200c induced reversal of EMT to MET phenotype (Fig. 4A). More importantly, re-expression of miR-200b and miR-200c significantly inhibited the prostasphere-forming ability of PC3 PDGF-D cells (Fig. 4B). Moreover, the size of prostaspheres was also reduced in PC3 PDGF-D cells transfected with miR-200b and miR-200c compared to transfection with control miRNA (Fig. S2A and S2B). However, transfection of PC3 PDGF-D cells with miR-200a had no effect on prostasphere-forming ability but increased the size of prostaspheres compared to control (Fig. 4B, Fig. S2A and Fig. S2B). These results suggest that miR-200b and miR-200c but not miR-200a inhibited self-renewal of PC3 PDGF-D cells by controlling target gene expressions of miR-200b and miR-200c. Thus, we assessed the expression of the putative targets of miR-200b and miR-200c such as Notch1, Bmi1 and lin28B in PC3 PDGF-D cells transfected with miR-200 family because miR-200b and miR-200c have three or one putative binding sites in the 3′UTR of Lin28B, Bmi1 mRNA (Fig. S3) and Notch1 mRNA. The miR-200b and miR-200c transfection dramatically reduced the expression of Notch1 and lin28B, but had no effect on the expression of Bmi1 (Fig. 4C). The expression of ZEB1 was found to be down-regulated by forced expression of miR-200b and miR-200c (Fig. 4C), which was consistent with the reversal of EMT phenotype (Fig 4A). We also found that miR-200c expression was repressed in ARCaP_M_ cells compared with ARCaP_E_ cells (Fig. 4D). Moreover, re-expression of miR-200c in ARCaP_M_ cells by transfection with pre-miR-200c precursors markedly reduced the prostasphere-forming capacity compared to transfection with control miRNA (Fig. 4E). These results were consistent with down-regulation of Notch1 expression and reversal of EMT phenotype as characterized by increased expression of E-cadherin and decreased expression of ZEB1 as well as cellular morphology change in ARCaP_M_ cells transfected with pre-miR-200c precursors (Fig. 4F and Fig. 4G).

### Down-regulation of Notch1 by miR-200 was partially responsible for the inhibition of colonogenic and prostasphere-forming ability of PC3 PDGF-D cells

**Figure 5:**
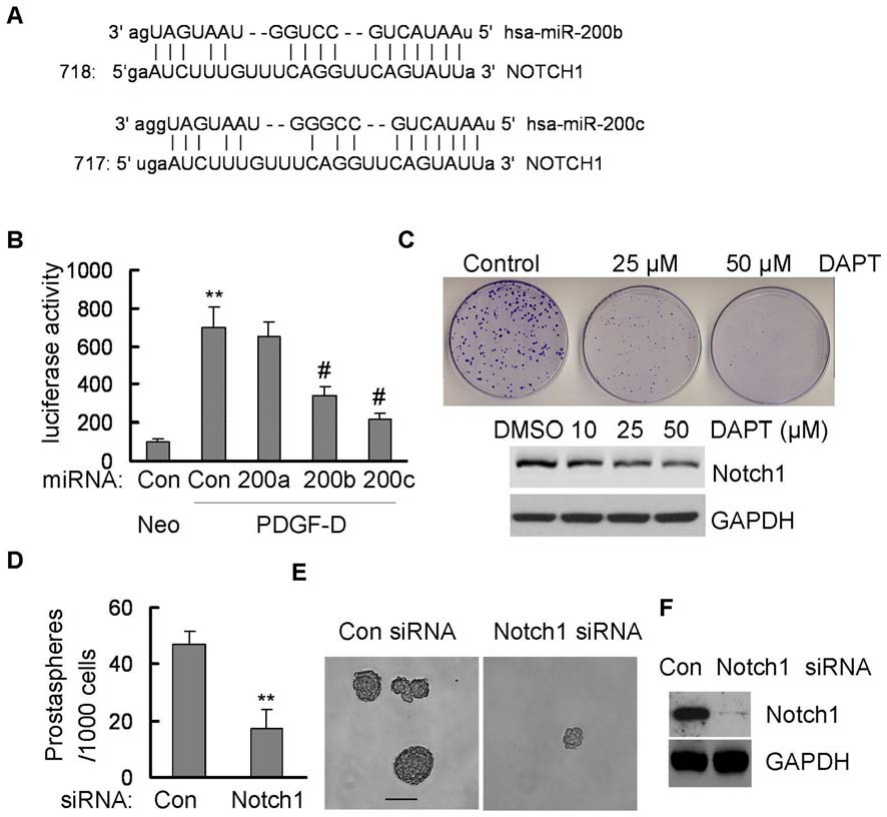
Inactivation of Notch1 repressed the colonogenic and prostasphere-forming capacity. (A) Conserved, predicted binding sites for the seed sequences of miR-200b and mir-200c in the 3′UTR of Notch1 mRNA. (B) MiR-200b and miR-200c inhibited the Notch1 3′UTR luciferase activity. **, p<0.01 compared to Neo control cells; #, p<0.01 compared to PDGF-D control cells. (C) DAPT treatment, a γ-secretase inhibitor, reduced the clonogenic ability in PC3 PDGF-D cells (upper panel).Western Blot showing that DAPT treatment reduced active form of Notch1 in dose-dependent manner (lower panel). (D) and (E) showing that transfection of Notch1 siRNA after 9 days repressed the prostasphere-forming capacity in PC3 PDGF-D cells (Bar:100 µm), which is consistent with downregulation of Notch1 expression (F). **, p<0.01 compared to control. (Neo: PC3 Neo cells, PDGF-D: PC3 PDGF-D cells, Con: control, 200a: pre-miR-200a, 200b: pre-miR-200b, 200c: pre-miR-200c).

The miR-200b and miR-200c has one binding sites in the 3′UTR of Notch1 (Fig. 5A). To determine whether Notch1 is the direct target of miR-200b and miR-200c, we analyzed the activity of 3′ UTR of Notch1 in PC3 Neo and PC3 PDGF-D cells transfected with 3′UTR of Notch1 luciferase plasmid as well as PC3 PDGF-D cells co-transfected with 3′UTR of Notch1 luciferase plasmid and miR-200b or miR-200c. We found that the activity of 3′UTR of Notch1 luciferase was significantly increased in PC3 PDGF-D cells compared with PC3 Neo cells due to lower levels of miR-200b and miR-200c in PC3 PDGF-D cells. Moreover, re-expression of miR-200b or miR-200c dramatically reduced the activity of 3′UTR of Notch1 luciferase in PC3 PDGF-D cells, while re-expression of miR-200a with one base of seed sequence different from miR-200b and miR-200c had no effects on the activity of 3′UTR of Notch1 luciferase (Fig. 5B). These results suggest that the miR-200b and miR-200c regulate the Notch1 expression by binding to 3′UTR of Notch1 mRNA. To determine whether Notch1 played an important role in maintaining the stem cells, we treated the PC3 PDGF-D cells with DAPT, a γ-secretase inhibitor that inhibits the activation of Notch. Full length Notch1 is usually very low due to its cleavage by many enzymes in these cells; however, we found that the expression of full length Notch1 was slightly more in γ-secretase treated cells. We also found that DAPT significantly reduced clonogenic ability of PC3 PDGF-D cells (Fig. 5C, upper panel), which was consistent with Western blot data showing that DAPT treatment inhibited active form of Notch1 (Fig. 5C, lower panel). Moreover, transfection of Notch1 siRNA dramatically repressed the prostasphere-forming ability of PC3 PDGF-D cells (Fig. 5D and 5E), which was consistent with down-regulation of Notch1 protein expression (Fig. 5F). These results suggest that miR-200 mediated down-regulation of Notch1 was partially responsible for self-renewal capacity and colonogenic growth of EMT-like cells having stem cell features.

### Lin28B inhibited production of let-7, regulating self-renewal by controlling the expression of Sox2, Nanog and Oct4

**Figure 6:**
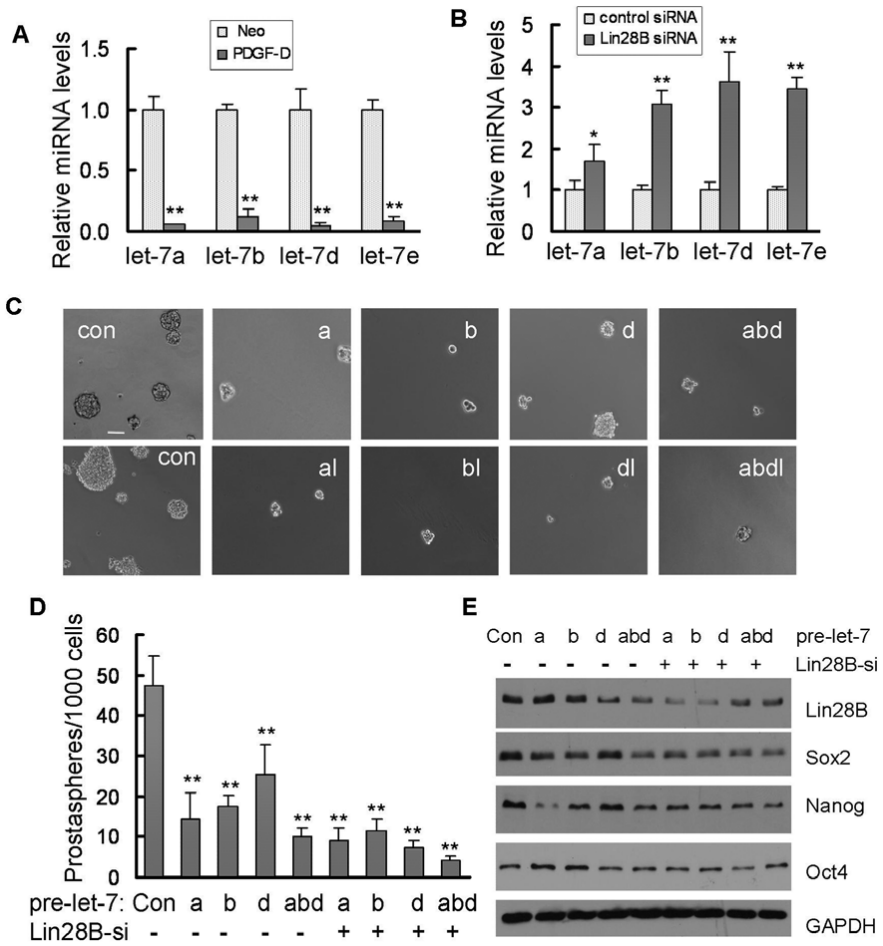
Lin28B-mediated repression of let-7 was partially responsible for regulating self-renewal in PC3 PDGF-D cells. (A) The levels for let-7 family in PC3 Neo and PC3 PDGF-D cells were determined by using real time RT-PCR. (B) Real time RT-PCR was used to quantify the expression of let-7 in PC3 PDGF-D cells transfected with Lin28B siRNA for 3 days. (C) Photomicrographs showing prostaspheres generated from PC3 PDGF-D cells transfected with pre-let-7 or combination of pre-let-7 and Lin28B siRNA 14 days after transfection (bar:100 µm). (D) The cells were collected for prostasphere-forming assay after 14-day transfection. Let-7a, let-7b, let-7d and combination of Let-7a, let-7b, let-7d as well as co-transfection with let-7 and Lin28B siRNA decreased the number of prostaspheres. (E) The results from Western blot showed the expression of Lin28B, Sox2, Nanog, and Oct4 in PC3 PDGF-D cells transfected with pre-let-7 or co-transfected with pre-let-7 and Lin28B siRNA after 9-day transfection. *, p<0.05 compared to control; **, p<0.01 compared to control. (Con: control, a: pre-let-7a, abd: combination of pre-let-7a, pre-let-7a, pre-let-7d, al: combination of pre-let-7a and Lin28B siRNA, Lin28B-si: Lin28B siRNA, Neo: PC3 Neo cells, PDGF-D: PC3 PDGF-D cells).

Our results presented above showed that the miR-200b and miR-200c inhibited lin28B expression (Fig. 4C), which is known to bind to primary let-7 and pre-let-7 RNA and inhibits the accumulation of mature let-7 miRNA [19]. The results from our miRNA microarray data showed that all the members of let-7 family were significantly reduced in PC3 PDGF-D cells (Table S3). These results were confirmed by real time RT-PCR assay (Fig. 6A). Knock-down of Lin28B by siRNA strongly increased mature let-7 expressions (Fig. 6B); suggesting lin28B regulated mature let-7 expression in PC3 PDGF-D cells. It is well known that let-7 family plays a critical role in regulating self-renewal in embryonic stem cells and breast cancer stem-like cells [18], [37]. To determine whether let-7 could control self-renewal of PC3 PDGF-D cells, we performed the sphere-forming assay. Re-expression of selected let-7 family such as let-7a, let-7b, let-7d or combination of let-7a, let-7b and let-7d markedly inhibited prostasphere-forming capability (Fig. 6C and 6D) and simultaneously reduced the size of prostaspheres (Fig. 6C, S4A and S4B). Since Lin28B inhibits accumulation of mature let-7 by binding to pre-let-7 RNA and thereby blocking the processing of pre-let-7 RNA, PC3 PDGF-D cells with high level of Lin28B were co-transfected with pre-let-7 and Lin28B siRNA. We found that prostasphere numbers from cells co-transfected with pre-let-7 and Lin28B siRNA were less than that of transfection with pre-let-7 alone (Fig. 6D); however, except for let-7d, there was no difference in prostasphere size between pre-let-7 alone and co-transfection with pre-let-7 and Lin28B siRNA (Fig. 6C, S4A and S4B). These results were consistent with the data from Western blot analysis showing that the re-expression of let-7 significantly inhibited Sox2 and Nanog expression in PC3 PDGF-D cells by transfection of pre-let-7a, pre-let-7b or co-transfection of pre-let-7a, pre-let-7b or pre-let-7d with Lin28B siRNA. Transfection of pre-let-7d alone was able to inhibit the expression of Lin28B and Oct4 but had marginal effects on Sox2 and Nanog expression (Fig. 6E).

### Sox2, Nanog, Oct4 and Lin28B were required for self-renewal of PC3 PDGF-D cells

**Figure 7:**
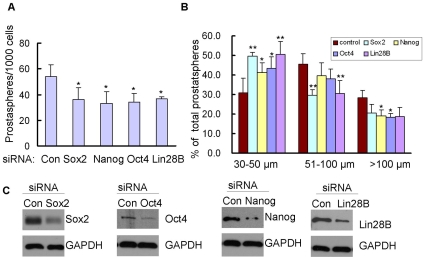
Sox2, Nanog, Oct4 and Lin28B regulated Prostasphere-forming ability of PC3 PDGF-D cells. (A) Single cell suspensions of PC3 PDGF-D transfected with Sox2, Nanog, Oct4 and Lin28B siRNA and incubated for 24 h were plated on ultra low adherent wells of 6-well plate at 2000 cells/well. After 3 days, the numbers of prostaspheres were counted under microscope. Transfection of Sox2, Nanog, Oct4 or Lin28B siRNA reduced the prostasphere-forming capacity (B) Prostaspheres were photographed and the size of prostaspheres in diameter was measured using software image analysis program Scion Image. (C). Western blot analysis showed that levels of Sox2, Nanog, Oct4 and Lin28B in PC3 PDGF-D cells transfected with Sox2, Nanog, Oct4 and Lin28B siRNA compared with transfection with control siRNA (*, p <0.05, **, p<0.01 compared to control, Con: control).

We have shown that let-7 family regulated self-renewal and inhibited Sox2, Nanog, Oct4 and Lin28B expression in PC3 PDGF-D cells with EMT phenotype. In order to assess the mechanistic link of these molecules (Sox2, Nanog, Oct4 and Lin28B) with the self-renewal capacity of PC3 PDGF-D cells, we knocked down the expression of Sox2, Nanog, Oct4 and Lin28B by siRNA transfection using Sox2, Nanog, Oct4 and Lin28B specific siRNA. We found that knock-down of Sox2, Nanog, Oct4 and Lin28B significantly repressed prostasphere-forming ability (Fig. 7A). Furthermore, PC3 PDGF-D cells transfected with Sox2, Nanog, Oct4 and Lin28B siRNA showed significantly increased numbers of prostaspheres in smaller size (30–50 µm) compared to that transfected with control siRNA concomitant with decreased numbers of prostaspheres with >50 µm in diameter (Fig. 7B). These results were consistent with protein expression results as shown in Fig. 7C, suggesting that Sox2, Nanog, Oct4 and Lin28B are required for self-renewal of PC3 PDGF-D cells.

### The cells with EMT phenotype promoted tumorigenicity

**Figure 8:**
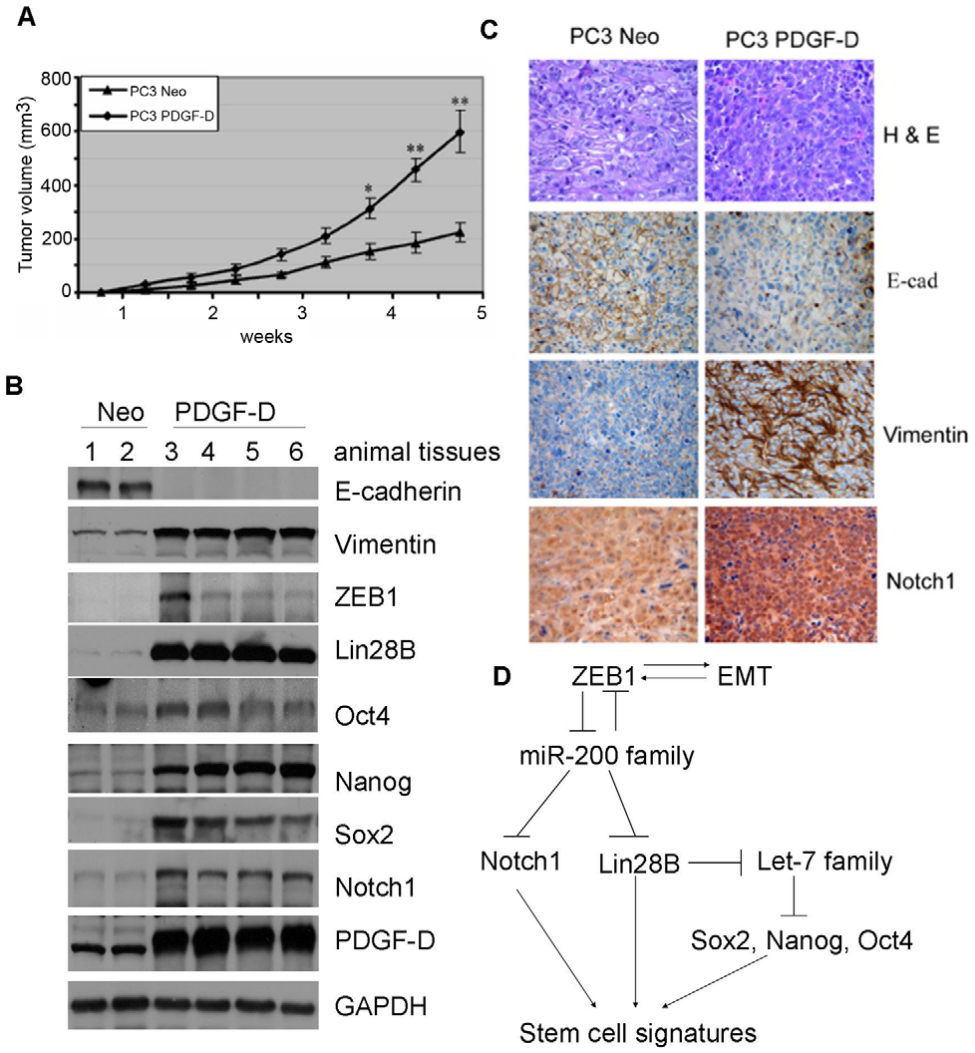
The cells with EMT phenotype promoted tumor growth. (A) Tumor growth curve showing that over-expression of PDGF-D could promote tumor growth in SCID mice much faster than PC3 Neo cells. (B) Western blot analysis of tumor lysates showing the expression of EMT and stem cell makers. (C) H&E evaluation of the tumors from both the groups showed high grade carcinoma associated with tumor apoptosis and necrosis. The results from the immunostaining showed much intense staining for vimentin and Notch1 and less intense staining for E-cadherin. (D) Regulatory model showing mechanistic link between EMT and stem cells: EMT induced by different factors characterized by increased expression of ZEB1, which causes loss of miR-200 family. Loss of miR-200b and miR-200c lead to increased ZEB1, Notch1 and Lin28B expression, resulting in decreased expression of let-7. Downregulation of let-7 leads to the up-regulation of Sox2, Nanog and Oct4, which together with Notch1 and Lin28B contribute to stem cell signatures. (*, p <0.05, **, p<0.01 compared to PC3 Neo, E-cad: E-cadherin).

In our previous studies, we showed that the tumors from PC3 PDGF-D cells grew much faster compared to that from PC3 Neo cells in an animal model of experimental bone metastasis [26]. For our current animal model studies, PC3 Neo and PC3 PDGF-D cells were injected subcutaneously into the right and left flanks of each mouse and compared tumor growth rate between PC3 Neo and PC3 PDGF-D cells. We found that the volume of tumors induced by PC3 PDGF-D cells was significantly larger than that of PC3 Neo cells (Fig. 8A). To reveal whether tumor cells i*n vivo* have the signatures in the expression of genes similar to *in vitro* experiments, we assessed the expression of selected genes related to EMT and stem cell markers. We found down-regulation of E-cadherin and up-regulation of mesenchymal markers such as vimentin and ZEB1 in tumor tissues derived from PC3 PDGF-D cells compared with PC3 Neo control cells. Concomitantly, Lin28B, Sox2, Nanog and Oct4 as well as Notch1 expression was also significantly increased in tumors derived from PC3 PDGF-D cells, which was consistent with increased PDGF-D expression (Fig. 8B). Immunohistochemical analysis showed that the tumors derived from PC3 PDGF-D cells contained much intense staining for vimentin and Notch1 and less intense staining for E-cadherin (Fig. 8C). These results suggest that the cells with EMT phenotype promoted tumorigenicity *in vivo.*

Taken together, EMT cells such as PC3 PDGF-D and ARCaP_M_ cells shared stem-like cell signatures characterized by increased colonogenic and prostasphere-forming capacity. The miR-200, especially miR-200b and miR-200c were identified to be mechanically linked with EMT phenotype and stem cell signatures in these cells via regulation of Notch1 and/or Lin28B expression. Lin28B inhibited accumulation of mature let-7, which resulted in the increased expression of Sox2, Nanog and Oct4 (Fig. 8D), leading to increased self-renewal and tumor growth. Thus, miR-200b and miR-200c appear to play a central role in linking EMT with stem cell features in prostate cancer progression.

## Discussion

Epithelial to mesenchymal transition is a physiological process during embryonic development and functions in adults to promote organ morphogenesis and tissue regeneration as well as wound healing. Emerging evidence suggest that the acquisition of EMT is strongly associated with cancer cell invasion and tumor metastasis. Recently, studies have shown that cells with EMT phenotype share characteristics that are consistent with the signatures of cancer stem-like cells, which are associated with tumor recurrence and drug resistant phenotype and contribute to the demise of patients diagnosed with cancers [12], [22], [38]. In the current study, we found that over-expression of PDGF-D in PC3 cells induced cellular morphological changes that were consistent with the acquisition of EMT phenotype as characterized by the loss of expression of epithelial markers and the gain or increased expression of mesenchymal markers. More importantly, PC3 PDGF-D cells displayed an increased clonogenic and prostasphere-forming capacity, the characteristics that are known to be associated with cancer stem-like cell characteristics. ARCaP_M_ cells were derived from ARCaP cells showing mesenchymal morphology and increased expression of mesenchymal markers such as ZEB1 and vimentin as well as decreased expression of epithelial markers such as E-cadherin. Interestingly, ARCaP_M_ cells also displayed stem-like cell phenotype characterized by increased clonogenic and prostasphere-forming capacity compared with ARCaP_E_ cells having epithelial phenotype. Our results suggest that EMT-type cells show stem-like cells or cancer stem-like cell characteristics.

It is well known that co-expression of Sox2, Nanog, Oct4 and Lin28 or Oct4, Sox2, Klf4 and c-myc in human or mouse somatic cells can reprogram these somatic cells into pluripotent embryonic stem-like cells [4], [5], [39]. These results are consistent with data from Dr. Ben-porath, et al. who demonstrated that histologically poorly differentiated tumors showed preferential over-expression of Nanog, Oct4, Sox2 and c-Myc genes that are normally enriched in embryonic stem cells (ES cells). Moreover, they also found that activation targets of Nanog, Oct4, Sox2 and c-Myc were more frequently over-expressed concomitant with repression of polycomb-regulated genes in poorly differentiated tumors than in well-differentiated tumors [7]. However, high-grade tumors with ES signatures failed to demonstrate CD44^high^/CD24^low^ population of cells in these tumors [7], suggesting the complexity of stem cell characteristics. In our study, we found that the expressions of Sox2, Nanog, Oct4 and Lin28B were dramatically up-regulated in PC3 PDGF-D cells. More importantly, knock-down of Sox2, Nanog, Oct4 and Lin28B by siRNA transfection repressed prostasphere-forming capacity of PC3 PDGF-D cells. Moreover, we also found enhanced expression of Stat3, Sall2, Sall4, Zic2 and Zic3, downstream targets of Nanog, Oct4 and Sox2, and up-regulation of EZH2, Suz12 and EED (which consisted polycomb repressor complex 2) in PC3 PDGF-D cells. Polycomb repressor complex 2 occupies developmental genes in ES cells to maintain pluripotency of ES cells. Target genes of polycomb repressor complex are known to be repressed in ES cells, and activated during the ES cell differentiation, and are regulated by Nanog, Oct4 and Sox2 [32], [33]. Taken together, PC3 PDGF-D cells with EMT signatures showed stem-like cell characteristics through over-expression of Nanog, Oct4 and Sox2, Lin28B and activation of polycomb repressor complex 2.

The miR-200 family plays a critical role in mediating EMT phenotype induced by various factors including PDGF-D [28], [40], [41]. In order to investigate whether miR-200 family could contribute to the generation of cancer stem-like cell characteristics in ARCaP_M_ and PC3 PDGF-D cells by regulating the expression of Nanog, Oct4 and Sox2, Lin28B and other stem cell-associated makers, we have searched targets of miR-200 family in www.microRNA.org. We found that miR-200b and miR-200c have three binding sites at the 3′UTR of Lin28B mRNA, one in Notch1, Sox2 and Bmi1. Interestingly, miR-200b and miR-200c were significantly decreased in PC3 PDGF-D cells and miR-200c was down-regulated in ARCaP_M_ cells. Therefore, we analyzed the expression of Lin28B, Notch1 and Bmi1, and performed the sphere-forming assay using ARCaP_M_ and PC3 PDGF-D cells transfected with miR-200. We found that re-expression of miR-200b and miR-200c dramatically reduced the sphere-forming ability concomitant with decreased expression of Lin28B and Notch1 in PC3 PDGF-D cells. The miR-200c also dramatically reduced the sphere-forming ability concomitant with decreased expression of Notch1 in ARCaP_M_ cells compared to its isogenic ARCaP_E_ cells.

Lin28B, a Lin28 homolog, was significantly repressed by re-expression of miR-200b and miR-200c in our cell model. It has been shown to selectively block the processing of pri-let-7 miRNAs in embryonic cells, resulting in decreased mature let-7 [19]. In the present study, we found that Lin28B was significantly increased and let-7 family was strongly repressed in PC3 PDGF-D cells. Transfection of Lin28B siRNA enhanced let-7 expression, suggesting increased Lin28B was responsible for the loss of let-7 in PC3 PDGF-D cells. The let-7 was known to promote differentiation of embryonic cells, reduced expression of let-7 was associated with increased self-renewal ability in breast cancer cells [18]. Thus, we hypothesized that let-7 could regulate self-renewal by regulating Nanog, Oct4, Sox2 and Lin28B expression in PC3 PDGF-D cells. In order to prove our hypothesis, PC3 PDGF-D cells were transfected with let-7, and we found that let-7a and let-7b could reduce the sphere-forming capacity concomitant with decreased expression of Sox2 and Nanog, These results were consistent with the data showing that let-7 significantly inhibited Oct4, Nanog and Sox2 expression in mouse embryonic stem cells [42].

In summary, the findings reported in this study showed, for the first time, that ARCaP_M_ and PC3 PDGF-D cells having EMT phenotype shared cellular and molecular characteristics of stem cells or cancer stem-like cells. Moreover, miR-200 and let-7 played a critical role in linking EMT phenotype with stem cell signatures by regulating the expression of Lin28B and Notch1. Therefore, we believe that these models would be useful in screening drug libraries for finding newer agents that could be useful in selective killing of EMT-type or cancer stem-like cells in prostate cancer in the future consistent with a similar approach that showed a great success in breast cancer [43]. Interestingly, recent studies have shown that some natural agents could up-regulate miR-200 and reverse the EMT phenotype [44], [45]. Thus, in future studies we will use these natural agents to eliminate the stem-like cells by reversing EMT phenotypic cells to MET phenotype for assessing whether natural agents could be useful for the prevention of prostate cancer recurrence and metastasis. We firmly believe that strategies by which one could either reverse the EMT to MET phenotype or could selectively kill EMT-phenotypic cells or cancer stem-like cells would become a novel approach for the prevention of tumor progression and/or treatment of CRPC and its metastasis for which newer therapies are urgently needed.

## Materials and Methods

### Ethics Statement

This study was carried out in strict accordance with the recommendations in the Guide for the Care and Use of Laboratory Animals of the National Institutes of Health. Any animal found unhealthy or sick were promptly euthanized. The protocol was approved by the Committee on the Ethics of Animal Experiments of Wayne State University institutional Users Animal Care Committee (Permit Number: A-10-03-08).

### Cell lines and culture condition

Generation of stable cell lines over-expressing PDGF-D was accomplished by transfection of PC3 cells with the corresponding empty vector pcDNA3 Neo or pcDNA3-PDGF-D:His as previously described [31] and referred to as PC3 Neo, or PC3 PDGF-D cells, respectively throughout this manuscript. The PC3 Neo or PC3 PDGF-D cell lines were cultured in RPMI 1640 medium with 2 mmol/L glutamine, 25 µmol/L Hepes (Invitrogen, Carlsbad, CA) supplemented with 5% fetal bovine serum (FBS), 50 units/ml Penicillin, and 50 µg/ml Streptomycin. ARCaP_E_ and ARCaP_M_ cells were purchased from Novicure Biotechnology (Birmingham, AL). ARCaP_E_ and ARCaP_M_ were established from bone tumor tissue derived from ARCaP cells. ARCaP_E_ with cobblestone morphology expressed higher E-cadherin and cytokeritin 18, 19, which were associated with typical epithelial cell characters, and concomitant with lower level of vimentin expression. In contract, ARCaP_M_ with spindle-shape fibrobastic morphology expressed more genes associated with mesenchymal markers such as increased vimentin and N-cadherin expression, and these cells also acquired drug-resistant phenotype [46]. These cells were cultured in Prostate Epithelial Cell Medium (MCaP) supplied by Novicure Biotechnology, and the medium was supplemented with 5% fetal bovine serum (FBS), 50 units/ml Penicillin, and 50 µg/ml Streptomycin. All cells were maintained in a 5% CO_2_-humidified atmosphere at 37°C, and genotypically characterized to support the authenticity of these cells, which was consistent with its origin.

### Research reagents and antibodies

Antibodies against slug, Oct4, Sox2, Nanog and Lin28B were purchased from Cell Signaling Technology (Beverly, MA). Antibody against EZH2 was purchased from BD Biosciences (Bedford, MA). Antibody to vimentin was acquired from Abcam (Cambridge MA). Antibodies against ZEB1, ZEB2, Stat3, E-cadherin, jagged2, delta1, delta3, delta4, Notch1, Notch2, and Notch3 were obtained from Santa Cruz (Santa Cruz, CA). Goat anti-rabbit IgG (H + L)-HRP conjugate and goat anti-mouse IgG (H + L)-HRP conjugate were obtained from Bio-Rad (Reinach, BL,). Antibody to glyceraldehyde 3-phosphate dehydrogenase (GAPDH) was purchased from Affinity BioReagents (Golden, CO). DAPT (N-[N-(3,5-Difluorophenacetyl-L-alanyl)]-S-phenylglycine *t*-Butyl Ester) was purchased from EMB Bioscience (San Diego, California).

### Clonogenic assay

PC3 Neo, PC3 PDGF-D, ARCaP_E_ and ARCaP_M_ cells were collected after trypsinization, and re-suspended in the complete medium. Single cell suspensions were plated in regular 10 cm in diameter Petri dishes at the clonal density of 1,000 cells per dish. After 2–3 weeks of culture, colonies were fixed with 4% paraformaldehyde for 10 min, stained with crystal violet for additional 10 min, and washed with 1X PBS. The colonies were photographed. The colony numbers were counted using software image analysis program Scion Image downloaded from NIH website (http://www.scioncorp.com). Particle Analysis program was used for counting the colony numbers. Data was presented as relative colony number of PC3 Neo or ARCaP_E_ designated as unit value of one.

### Soft agar assay

PC3 Neo and PC3 PDGF-D cells were harvested and suspended in culture medium. To make the bottom layer, 1 ml of 0.5% agarose (Invitrogen) was added to 6-well plates, and allowed to gel at room temperature. To prepare the top layer (0.25% agarose), 500 ìl of 0.5% agarose was mixed with 500 ìl cell suspension containing the 5000 cells. This mixture were overlaid above the bottom layer and allowed to solidify at room temperature. An additional 2 ml of culture medium was added after solidification to the top layer, and cells were incubated for 3 weeks at 37°C. After three weeks of growth, the colonies were photographed (40 X). The colony numbers were counted under a phase contrast microscope (40 X). Data was presented as colony numbers per field.

### Self-renewal assay

Single cell suspensions of PC3 Neo, PC3 PDGF-D, ARCaP_E_, ARCaP_M_ and PC3 PDGF-D cells transfected with Sox2, Nanog, Oct4, Lin28B or control siRNA as well as miR-200 and let-7, were plated on ultra low adherent wells of 6-well plate (Corning, Lowell, MA) at 1000 or 2000 cells/well in DMEM/F12 (Invitrogen) supplemented with B27 and N2 (Invitrogen). Single cell status was confirmed under microscope. Fresh medium was added every 3–4 days. After 3 or 6 days, spheres termed here as “prostaspheres” (P1) numbers were counted under microscope and the proportion of sphere-generating cells was calculated by dividing the number of cells seeded by the number of prostaspheres. Prostaspheres were photographed and the size of the prostaspheres in diameter was measured using software image analysis program Scion Image downloaded from NIH website. To confirm that prostaspheres were the progeny of individual cells rather than the aggregation of the multiple cells, the prostaspheres (P1) were collected by centrifugation (300 X g for 5 min) and dissociated with accucute (Sigma) and the cells were plated at one cell per well in 96-well plate with ultra low attachment. Single cell status was confirmed under microscope. After 12 days incubation, the prostasphere (P2) numbers were counted under a phase contrast microscope.

### Western blot analysis

Total cell lysates from different experiments were obtained by lysing the cells in RIPA buffer containing 50 mM Tris–HCl, 150 mM NaCl, 1% NP-40, 0.1% SDS, 0.5% sodium deoxycholate, 2 mM sodium fluoride, 2 mM Na_3_VO4_2_, 1 mM EDTA, 1 mM EGTA, and 1 X protease inhibitor cocktail. Western blotting was performed as previously described [47].

### siRNA, miRNA and transfection experiments

Cells were transfected with 100 nmol/L of Sox2, Nanog, Oct4, Lin28B siRNA or control siRNA (Santa Cruz) as well as 20 nmol/L of miR-200 or let-7 (Ambion, Austin, TX) using DharmaFECT3 siRNA transfection reagent (DHARMACON, Lafayette, CO). After 3 days of transfection, cells were split and transfected repeatedly with siRNA or miRNA every 3–4 days for indicated times. After 24 h of incubation from last transfection, the cells were collected after trypsinization and then re-suspended in DMEM/F12 (Invitrogen) supplemented with B27 and N2 (Invitrogen). The single cell suspensions were plated on ultra low adherent wells of 6-well plate (Corning) at 2000 cells/well for generation of prostaspheres. In addition, the cell lysates from cells transfected with siRNA or pre-miRNA were prepared for Western blot analysis.

### Luciferase activity assay

PC3 Neo and PC3 PDGF-D cells were seeded at a density of 6×10^3^ cells per well in 96-well plate and incubated for 24 h. The cells were co-transfected with Notch1 3′UTR luciferase plasmid (Switch Gear Genomics, Menlo Park, CA) and pre-miR-200 using DharmaFECT duo transfection reagent (DHARMACON, Lafayette, CO). After 48 h of transfection, luciferase activity was assayed using Steady–Glo Luciferase Assay System (Promega). The renilla luciferase activity was used as a control for transfection efficiency.

### Real-time RT-PCR

The total RNA was isolated using the Trizol reagent. Two micrograms of RNA were transcribed into cDNA using High Capacity RNA-to-cDNA Kit (Applied biosystems, Fostor, CA) according to the manufacturer's instruction. Real time PCR was used to quantify mRNA expression by using SYBR® Green RT-PCR Reagents (Applied biosystems). Sequences of primers were shown in table S4. The relative amount of RNA was normalized to the expression of GAPDH. For miRNA assay, the total RNA was isolated and PCR was performed as previously described [28].

### Microarray for gene profiling and miRNA microarray

Microarray and miRNA microarray as well as data analysis were conducted following procedures documented under supplementary materials and methods in text S1. All data is MIAME compliant and that the raw data has been deposited in a MIAME compliant database. GEO accession number is GSE22764.

### Tumor growth in SCID mice

Male homozygous CB-17 SCID/SCID mice (8 weeks old) were purchased from Taconic Farms (Germantown, NY). The mice were maintained according to the Animal Care and Use Guidelines approved by the NIH. Mice received Lab Diet 5021 (Purina Mills, Inc., Richmond, IN). After 1 week of acclimatization, the mice were randomized into two groups (n = 5) for our study. PC3 Neo and PC3 PDGF-D cells were harvested and re-suspended in serum-free RPMI medium. Cells (1×10^5^) in 100 µl serum-free RPMI medium were injected subcutaneously into the right and left flanks of each mouse. As soon as the majority of the tumors began to enlarge (50–75 mm^3^ volume, as determined by caliper measurements, 15–20th day after cell injection), mice with tumor take rate of 100% were carefully monitored and tumor volume were calculated in each group by twice-weekly caliper measurements. Comparison of growth kinetics between the PC3 Neo and PC3 PDGF-D cell line using this model system, the body weight of mice in each group was also monitored. All mice were euthanized 5 weeks later after the initiation of the experiment because large tumors were formed in the PC3 PDGFD group of mice, which required termination and their final body weight and tumor volume was recorded.

On autopsy, the tumor was neatly excised free of any extraneous adhering tissue. The tissue was rapidly frozen in liquid nitrogen, and stored at −70°C and subsequently used for preparation of protein extracts. Total protein was extracted from tumor tissues and prepared for Western blot analysis. Randomly selected harvested tumor tissue from each group of experimental mice were homogenized using Dounce homogenizer (Kontes Co., Vineland, NJ) containing 0.3 ml of ice-cold RIPA buffer solution with added protease inhibitors and 0.1 mM PMSF. The suspension was centrifuged at 16,000× g at 4°C for 10 min. The supernatant was collected and kept at −70°C until used for Western blot analysis.

### Immunohistochemistry

Formalin-fixed tumor tissue sections were evaluated for tumor cell morphology, mitotic rate, growth pattern, necrosis, cystic change, and inflammatory cellular response. Immunohistochemical studies were performed after staining with specific primary antibodies against E-cadherin, Vimentin and Notch1, followed by 3,3′-diaminobenzidine (DAB). Sections were visualized under an Olympus microscope (Olympus, Japan) and images were captured with an attached camera linked to a computer.

### Data analysis

Experiments (in vitro) presented in the figures are representative of three or more independent repetitions. The data are presented as the mean values ± SD. Comparisons between groups were evaluated by a two-tailed student's *t* test. Values of p<0.05 were considered statistically significant.

## Supporting Information

Text S1 Supplementary materials and methods.
(0.04 MB DOC)

Figure S1 PDGF-D levels in PC3 Neo and PC3 PDGF-D cells. Western Blot showed full length and active form of PDGF-D from PC3 PDGF-D cell lysates (A) and conditioned medium (B).
(1.63 MB TIF)

Figure S2 MiR-200b and miR-200c inhibited prostasphere-forming ability of PC3 PDGF-D cells. PC3 PDGF-D cells were transfected with pre-miR-200. 3 days after transfection, cells were split and transfected repeatedly with pre-miR-200 every 3–4 days for 14 days. (A) MiR-200b and miR-200c increased the numbers of prostaspheres with <100 µm in diameter and decreased the numbers of prostaspheres with >100 µm in diameter. (B) MiR-200b and miR-200c but not miR-200a reduced the size of prostaspheres.
(1.66 MB TIF)

Figure S3 Binding sites of miR-200b and miR-200c in 3′UTR of Lin28B or Bmi1 mRNA. Conserved, predicted binding sites for the seed sequences of miR-200b and mir-200c in the 3′UTR of Lin28B or Bmi1 mRNA were shown.
(3.36 MB TIF)

Figure S4 Let-7 regulated the self-renewal of PC3 PDGF-D. (A) Transfection of pre-let-7 or combination of pre-let-7 and Lin28B siRNA reduced the size of prostaspheres. (B) Transfection of pre-let-7 or combination of pre-let-7 and Lin28B siRNA increased the numbers of prostaspheres with smaller size (30–50 µm) and reduced the number of prostaspheres with bigger size (>100 µm) compared to transfection with control. **, p<0.01 compared to control (Con: control, a: pre-let-7a, abd: combination of pre-let-7a, pre-let-7a, pre-let-7d, al: combination of pre-let-7a and Lin28B siRNA, Lin28B-si: Lin28B siRNA).
(1.75 MB TIF)

Table S1 Fold change in EMT-associated genes induced by over-expression of PDGF-D in PC3 cells.
(0.03 MB DOC)

Table S2 Fold change in the expression of genes associated with stem-like cells in PDGF-D over-expressing PC3 cells compared to PC3 Neo cells.
(0.04 MB DOC)

Table S3 The levels of miR-200 and let-7 family in PC3 Neo and PC3 PDGF-D cells.
(0.02 MB DOC)

Table S4 Primer sequences used in real time PCR.
(0.04 MB DOC)
