# Supplementary material for: Epithelial to Mesenchymal Transition Is Mechanistically Linked with Stem Cell Signatures in Prostate Cancer Cells
Source: PLoS One. 2010 Aug 27;5(8):e12445. doi: 10.1371/journal.pone.0012445 (PMC2929211; doi:10.1371/journal.pone.0012445)
Supplement: Text S1 — Supplementary materials and methods. (0.04 MB DOC) [file pone.0012445.s001.doc]

**Epithelial to Mesenchymal Transition is Mechanistically Linked with Stem Cell Signatures in Prostate Cancer Cells**

**Dejuan Kong*, Sanjeev Banerjee*, Aamir Ahmad, Yiwei Li, Zhiwei Wang, Seema Sethi and Fazlul H. Sarkar**

Department of Pathology, Karmanos Cancer Institute, Wayne State University School of Medicine, Detroit, Michigan

**All correspondence to:** Fazlul H. Sarkar, Department of Pathology, Barbara Ann Karmanos Cancer Institute, Wayne State University School of Medicine, 740 Hudson Webber Cancer Research Center, 4100 John R, Detroit, MI 48201. Tel: 313-576-8327; Fax: 313-576-8389; Email: fsarkar@med.wayne.edu

* Equal contribution by these authors.

**Supplementary materials and methods**

***RNA isolation and quality control for conducing gene expression profiling by microarray***

Total RNA from PC3 Neo and PC3 PDGF-D cells was isolated using Trizol reagent (invitrogen) and further purified using RNeasy Mini kit and RNase-free DNase Set (Qiagen, Valencia, CA) according to the manufacturer's instruction. RNA purity and integrity were evaluated by microfluidics analysis using Agilent 2100 bioanalyzer and all the RNA samples with 28S:18S ratio >1.8 and RNA integrity number (RIN) >9.7 were used for microarray analysis.

***RNA labeling, hybridization***

Generation of labeled cRNA was performed using Illumina Totalprep RNA Amplification Kit (Illumina, San Diego, CA) according to the manufacture’s instruction. First strand cDNA for each sample (500 ng of total RNA) was synthesized by reverse transcription with an oligo(dT) primer bearing a T7 promoter using Array-Script. The cDNA then undergoes second strand synthesis. The cDNA was purified to remove RNA, enzyme and salt and to become a template for in vitro transcription with T7 RNA Polymerase. Transcription was performed using biotin UTP to generate biotinylated cRNA. The labeled cRNA was used to HumanWG-6 v3.0 BeadChip (Illumina, San Diego, CA), which contains 48,000 human gene probes, and hybridized to the probes in the array. After washing and staining, the arrays were scanned using Illumina's BeadStudio Software suite (v 3.1, Illumina).

***Microarray raw data analysis***

The raw gene expression data was obtained from the array scanned images using Illumina's BeadStudio Software suite (v 3.1). Data was normalized using the quantile normalization method [1], in which the probe intensity distribution in each array was transformed to match one of the average array. The fold changes were computed between the different groups, and genes with a fold change greater than 2 in a given comparison were retained for subsequent analysis. An enrichment analysis was performed on the selected genes to identify biological processes, molecular functions, cellular component and KEGG pathways that were over-represented in the list of selected genes. GO terms or pathways with a False Discovery Rate [2] of less than 0.1 were considered significant. All analyses were performed using the R statistical software using specialized bioconductor packages including limma and Gostats [3].

***MicroRNA microarray***

The total RNA was isolated using mirVana miRNA isolation kit (Ambion) according to the manufacturer’s instruction. Microarray assay was performed using a service provider (LC Sciences).  The assay started from 2 to 5 µg total RNA sample, which was size fractionated using a YM-100 Microcon centrifugal filter (from Millipore) and the small RNAs (< 300 nt) isolated were 3’-extended with a poly(A) tail using poly(A) polymerase.  An oligonucleotide tag was then ligated to the poly(A) tail for later fluorescent dye staining; two different tags were used for the two RNA samples in dual-sample experiments. Hybridization was performed overnight on a µParaflo microfluidic chip using a microcirculation pump (Atactic Technologies). On the microfluidic chip, each detection probe consisted of a chemically modified nucleotide-coding segment complementary to target microRNA (from miRBase, http://microrna.sanger.ac.uk/sequences/) and a spacer segment of polyethylene glycol to extend the coding segment away from the substrate.  The detection probes were made by in situ synthesis using PGR (photogenerated reagent) chemistry.  The hybridization melting temperatures were balanced by chemical modifications of the detection probes. Hybridization used 100 µL 6xSSPE buffer containing 25% formamide at 34 °C.  After hybridization, detection used fluorescence labeling using tag-specific Cy3 and Cy5 dyes.  Hybridization images were collected using a laser scanner (GenePix 4000B, Molecular Device) and digitized using Array-Pro image analysis software (Media Cybernetics). Data were analyzed by first subtracting the background and then normalizing the signals using a LOWESS filter10 (Locally-weighted Regression).  For two color experiments, the ratio of the two sets of detected signals (log2 transformed, balanced) and p-values of the t-test were calculated; differentially detected signals were those with less than 0.01 p-values.

Reference

1. Bolstad BM, Irizarry RA, Astrand M, Speed TP (2003) A comparison of normalization methods for high density oligonucleotide array data based on variance and bias. Bioinformatics 19: 185-193.

2. Hochberg Y, Benjamini Y (1990) More powerful procedures for multiple significance testing. Stat Med 9: 811-818.

3. Falcon S, Gentleman R (2007) Using GOstats to test gene lists for GO term association. Bioinformatics 23: 257-258.
