# Supplementary figures and images for: Epithelial to Mesenchymal Transition Is Mechanistically Linked with Stem Cell Signatures in Prostate Cancer Cells
Source: PLoS One. 2010 Aug 27;5(8):e12445. doi: 10.1371/journal.pone.0012445 (PMC2929211; doi:10.1371/journal.pone.0012445)

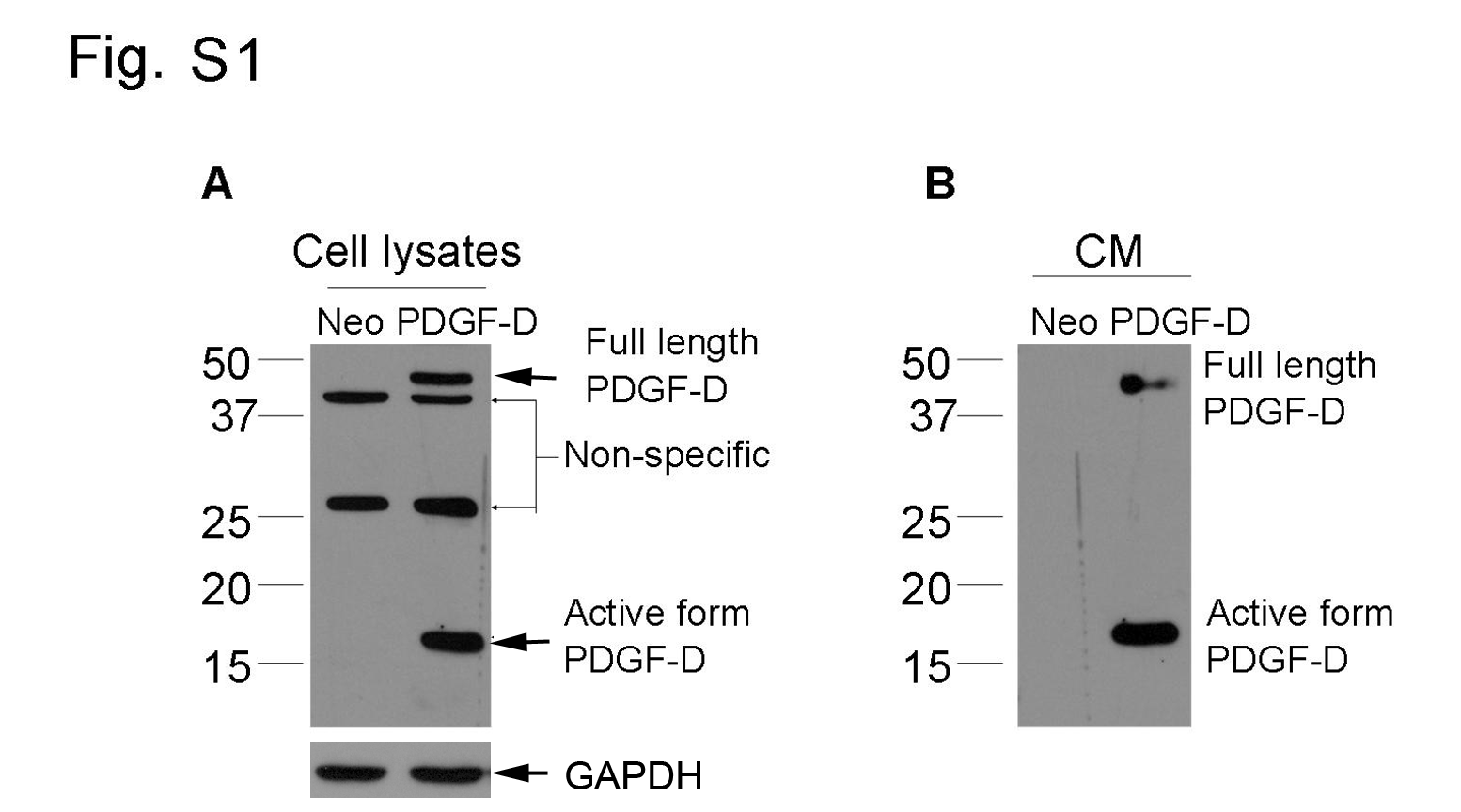

Supplement: Figure S1 — PDGF-D levels in PC3 Neo and PC3 PDGF-D cells. Western Blot showed full length and active form of PDGF-D from PC3 PDGF-D cell lysates (A) and conditioned medium (B). (1.63 MB TIF) [file pone.0012445.s002.tif]

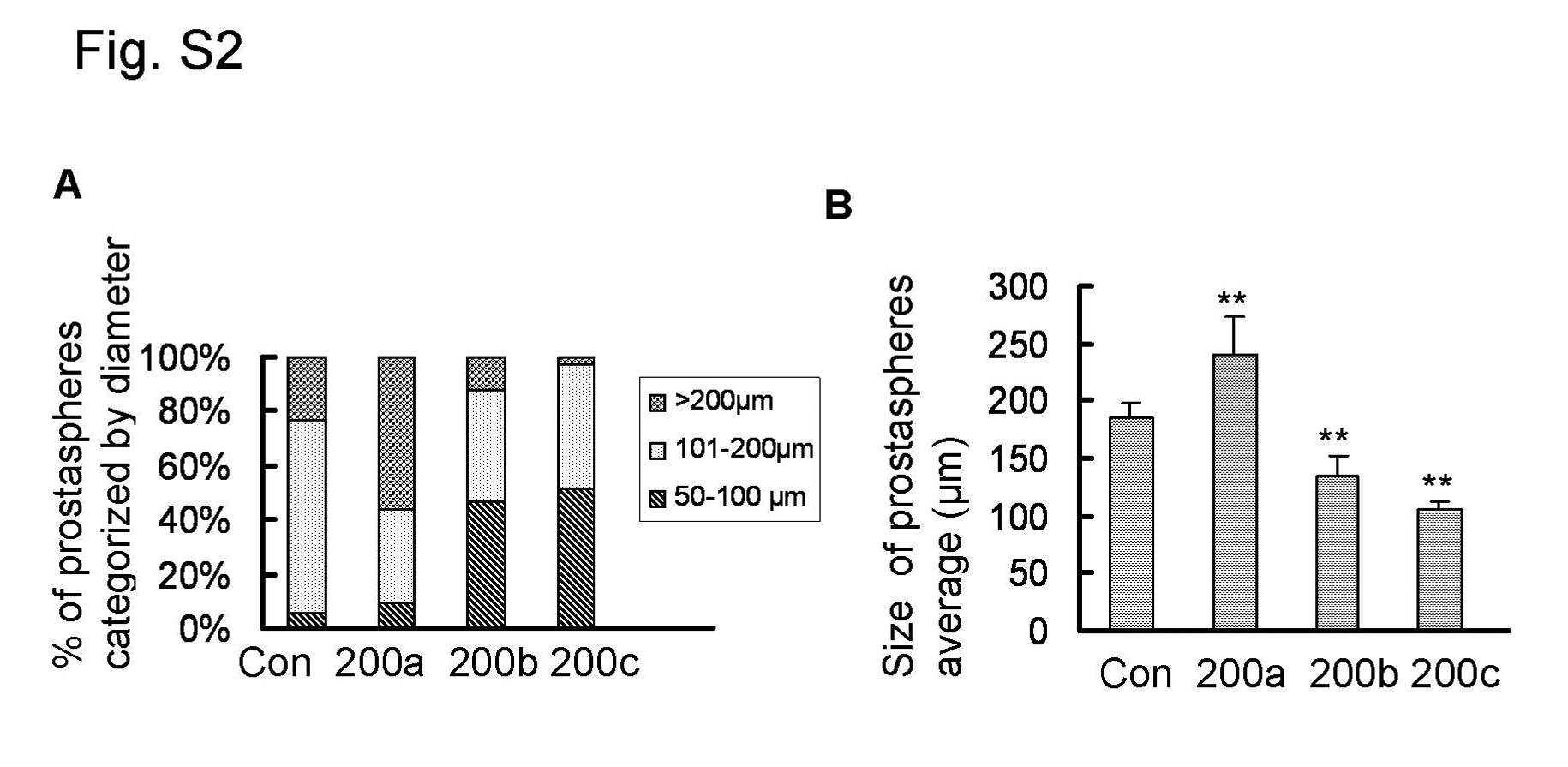

Supplement: Figure S2 — MiR-200b and miR-200c inhibited prostasphere-forming ability of PC3 PDGF-D cells. PC3 PDGF-D cells were transfected with pre-miR-200. 3 days after transfection, cells were split and transfected repeatedly with pre-miR-200 every 3–4 days for 14 days. (A) MiR-200b and miR-200c increased the numbers of prostaspheres with <100 µm in diameter and decreased the numbers of prostaspheres with >100 µm in diameter. (B) MiR-200b and miR-200c but not miR-200a reduced the size of prostaspheres. (1.66 MB TIF) [file pone.0012445.s003.tif]

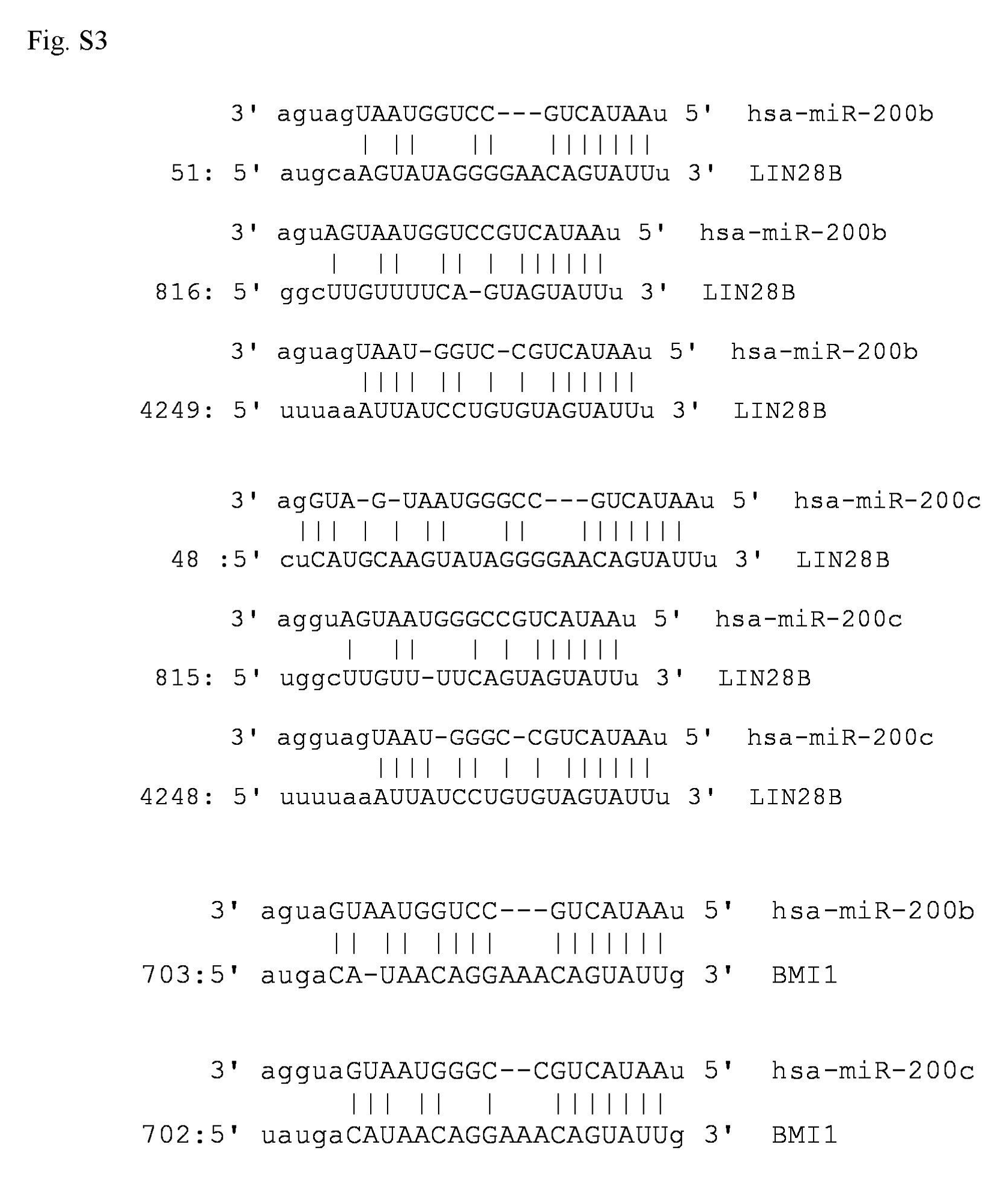

Supplement: Figure S3 — Binding sites of miR-200b and miR-200c in 3′UTR of Lin28B or Bmi1 mRNA. Conserved, predicted binding sites for the seed sequences of miR-200b and mir-200c in the 3′UTR of Lin28B or Bmi1 mRNA were shown. (3.36 MB TIF) [file pone.0012445.s004.tif]

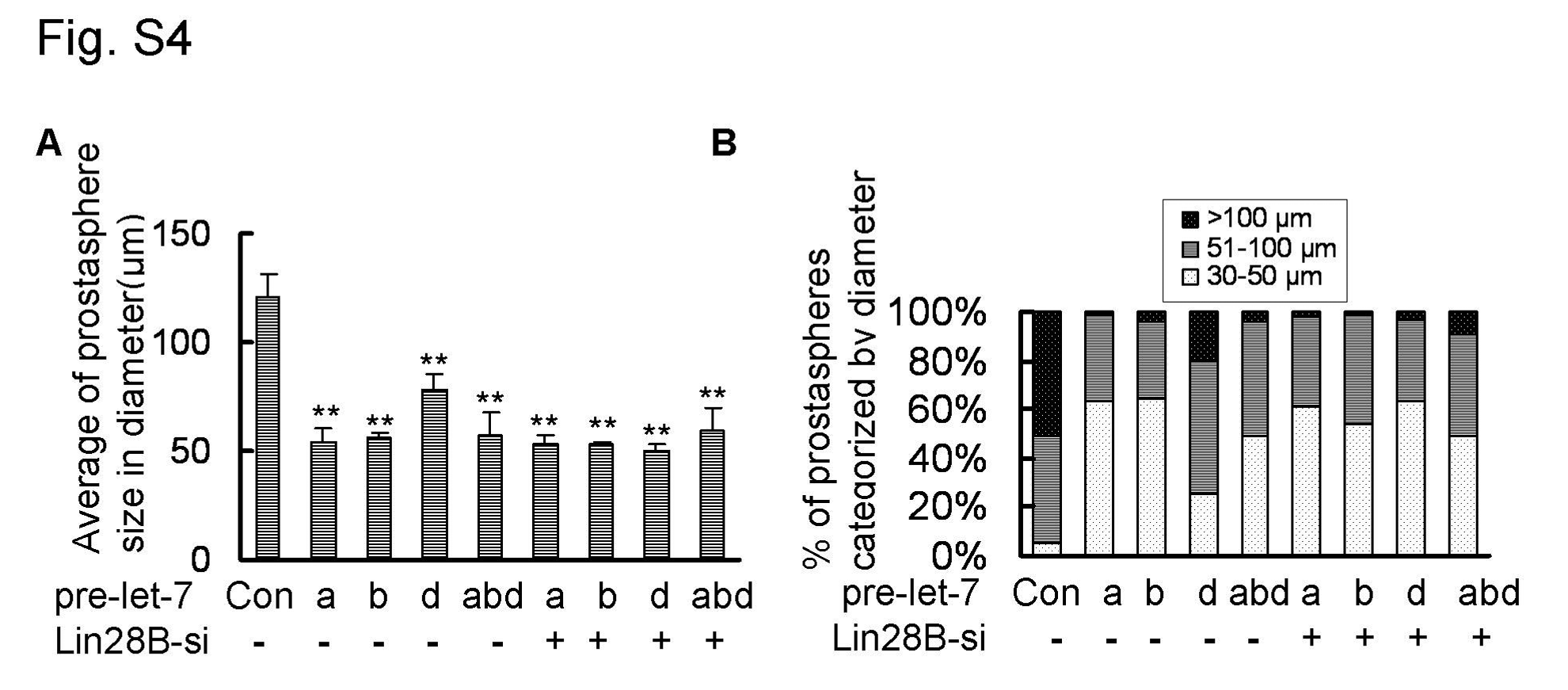

Supplement: Figure S4 — Let-7 regulated the self-renewal of PC3 PDGF-D. (A) Transfection of pre-let-7 or combination of pre-let-7 and Lin28B siRNA reduced the size of prostaspheres. (B) Transfection of pre-let-7 or combination of pre-let-7 and Lin28B siRNA increased the numbers of prostaspheres with smaller size (30–50 µm) and reduced the number of prostaspheres with bigger size (>100 µm) compared to transfection with control. **, p<0.01 compared to control (Con: control, a: pre-let-7a, abd: combination of pre-let-7a, pre-let-7a, pre-let-7d, al: combination of pre-let-7a and Lin28B siRNA, Lin28B-si: Lin28B siRNA). (1.75 MB TIF) [file pone.0012445.s005.tif]
