# Supplementary material for: Epithelial to Mesenchymal Transition Is Mechanistically Linked with Stem Cell Signatures in Prostate Cancer Cells
Source: PLoS One. 2010 Aug 27;5(8):e12445. doi: 10.1371/journal.pone.0012445 (PMC2929211; doi:10.1371/journal.pone.0012445)
Supplement: Table S1 — Fold change in EMT-associated genes induced by over-expression of PDGF-D in PC3 cells. (0.03 MB DOC) [file pone.0012445.s006.doc]

Table S1: Fold change in EMT-associated genes induced by over-expression of PDGF-D in PC3 cells

Symbol Name Fold-change

**Cadherins**

CDH1 Cadherin 1, type 1, E-cadherin (epithelial) -37.83

CDH3 Cadherin 3, type 3, P-cadherin (placental) -20.70

CDH11 Cadherin 11, type 2, OB-cadherin (osteoblast) -13.25

PCDH20 Protocadherin 20 9.13

PCDH7 Protocadherin 7 6.38

PCDH10 Protocadherin 10 5.81

PCDH19 Protocadherin 19 5.35

PCDH19 Protocadherin 18 4.54

CDH2 Cadherin 2, type 1, N-cadherin (neuronal) 2.25

**Cell apical and polarity genes**

CRB3 Crumbs homolog 3 (Drosophila) -20.31

PARD6G par-6 partitioning defective 6 homolog gamma (C. elegans) 5.78

PARD6A par-6 partitioning defective 6 homolog alpha (C. elegans) -3.71

LLgL2 Lethal giant larvae homolog 2 (Drosophila) -2.71

MUC1 mucin 1, cell surface associated -2.57

PARD3 par-3 partitioning defective 3 homolog (C. elegans) -2.06

DLG5 discs, large homolog 5 (Drosophila) -2.05

**Tight junctions**

CLDN11 claudin 11 (oligodendrocyte transmembrane protein) -47.66

CLDN1 claudin 1 -33.18

MARVELD3 MARVEL domain containing 3 -11.90

TJP1 tight junction protein 1 (zona occludens 1) -2.87

TJP3 tight junction protein 3 (zona occludens 3) -2.58

TJP2 tight junction protein 2 (zona occludens 2) -2.25

F11R F11 receptor -2.20

CLDN23 claudin 23 -2.11

CLDN4 claudin 4 -2.09

MARVELD2 MARVEL domain containing 2 -2.00

**Desmosomes and epidermis**

SFN stratifin -34.91

PPL periplakin -3.82

PKP3 plakophilin 3 -3.40

SH3YL1 SH3 domain containing, Ysc84-like 1 (S. cerevisiae) -2.94

SCEL sciellin -2.79

**Gap Junctions**

GJB3 gap junction protein, beta 3, 31kDa (connexin31) -19.73

GJA1 gap junction protein, alpha 1, 43kDa 12.15

GJB2 gap junction protein, beta 2, 26kDa (connexin26) -9.39

**Tubulins**

TUBB2B tubulin, beta 2B 96.81

TUBA4A tubulin, alpha 4a -35.29

TUBB3 tubulin, beta 3 -6.28

TUBB6 tubulin, beta 6 6.14

TUBB4 tubulin, beta 4 4.64

**Cell surface receptor**

TACSTD1 tumor-associated calcium signal transducer 1 (EpCAM) -121.735

TACSTD2 tumor-associated calcium signal transducer 2 - 80.54

TMEPAI transmembrane, prostate androgen induced RNA -15.49

**Vesicle transport**

MAL2 mal, T-cell differentiation protein 2 -69.09

TMEM30B transmembrane protein 30B -45.26

SYTL1 synaptotagmin-like 1 -3.50

**Transcription factors**

SNAI2 snail homolog 2 (Drosophila) 8.00

ZEB2 zinc finger E-box binding homeobox 2 2.25

ZEB1 zinc finger E-box binding homeobox 1 2.01

Twist twist homolog 1 1.56

SNAI1 snail homolog 1 (Drosophila) 1.03

**Cell skeleton**

LAMB3 laminin, beta 3 - 54.62

Vim vimentin 52.16

LAMC2 laminin, gamma 2 -26.72

LAMA3 laminin, alpha 3 -13.04

LAMC3 laminin, gamma 3 8.66

LAMA1 laminin, alpha 1 6.20

LAMA5 laminin, alpha 5 -2.69

**Keratins**

KRT81 keratin 81 -132.35

KRT19 keratin 19 -129.57

KRT6B keratin 6B -119.98

KRT7 keratin 7 -91.64

KRT80 keratin 80 -63.52

KRT75 keratin 75 -63.32

KRT18 keratin 18 -24.72

KRT8 keratin 8 -20.84

KRT17 keratin 17 -8.74

KRT34 keratin 34 -4.59

KRT15 keratin 15 -3.47

KRT86 keratin 86 -3.26

KRT 6A keratin 6A -3.26

**Integrin**

ITGB4 integrin, beta 4 -23.00

ITGB2 integrin, beta 2 (complement component 3 receptor 3 and 4 subunit) -15.66

ITGA2 integrin, alpha 2 (CD49B, alpha 2 subunit of VLA-2 receptor) -11.83

ITGB1 integrin, beta 1 (fibronectin receptor, beta polypeptide, -2.55

antigen CD29 includes MDF2, MSK12)

ITGA4 integrin, alpha 4 (antigen CD49D, alpha 4 subunit of VLA-4 receptor) 1.96

ITGA3 integrin, alpha 3 (antigen CD49C, alpha 3 subunit of VLA-3 receptor) -1.75
