# Supplementary material for: Epithelial to Mesenchymal Transition Is Mechanistically Linked with Stem Cell Signatures in Prostate Cancer Cells
Source: PLoS One. 2010 Aug 27;5(8):e12445. doi: 10.1371/journal.pone.0012445 (PMC2929211; doi:10.1371/journal.pone.0012445)
Supplement: Table S2 — Fold change in the expression of genes associated with stem-like cells in PDGF-D over-expressing PC3 cells compared to PC3 Neo cells. (0.04 MB DOC) [file pone.0012445.s007.doc]

Table S2: Fold change in the expression of genes associated with stem-like cells in PDGF-D over-expressing PC3 cells compared to PC3 Neo cells.

Symbol Name Fold-change

**Sox family and transcription factors**

Sox11 SRY (sex determining region Y)-box 11 109.80

Sox17 SRY (sex determining region Y)-box 17 53.69

Pou4F1 POU class 4 homeobox 1 38.03

LIN28B lin-28 homolog B (C. elegans) 24.58

Sox3 SRY (sex determining region Y)-box 3 18.26

Sox8 SRY (sex determining region Y)-box 8 18.05

STAT3 signal transducer and activator of transcription 3 15.20

(acute-phase response factor)

ZIC2 Zic family member 2 (odd-paired homolog, Drosophila) 11.82

SALL2 sal-like 2 (Drosophila) 11.37

Sox2 SRY (sex determining region Y)-box 2 7.81

Sox18 SRY (sex determining region Y)-box 18 7.75

Sox7 SRY (sex determining region Y)-box 7 -5.39

Sox15 SRY (sex determining region Y)-box 15 -5.13

Sox9 SRY (sex determining region Y)-box 9 -4.42

(campomelic dysplasia, autosomal sex-reversal)

Sox13 SRY (sex determining region Y)-box 13 -2.47

Sox21 SRY (sex determining region Y)-box 21 2.34

ZIC3 Zic family member 3 heterotaxy 1 (odd-paired homolog, 1.80

Drosophila)

SALL4 sal-like 4 (Drosophila) 1.63

Pou5F1 POU class 5 homeobox 1 1.15

**Polycomb group of proteins**

SUZ12 suppressor of zeste 12 homolog (Drosophila) 1.73

EZH2 enhancer of zeste homolog 2 (Drosophila) 1.71

EED embryonic ectoderm development 1.23

**Wnt signaling**

DKK1 dickkopf homolog 1 (Xenopus laevis) -43.20

RAC2 ras-related C3 botulinum toxin substrate 2 -42.23

(rho family, small GTP binding protein Rac2)

FOSL1 FOS-like antigen 1 -41.36

FZD9 frizzled homolog 9 (Drosophila) 20.96

WNT3A wingless-type MMTV integration site family, member 3A 14.35

AXIN2 axin 2 (conductin, axil) 9.56

WNT7A wingless-type MMTV integration site family, member 7A -9.38

SFRP1 secreted frizzled-related protein 1 8.58

ROR2 receptor tyrosine kinase-like orphan receptor 2 7.04

NKD2 naked cuticle homolog 2 (Drosophila) 5.04

GSK3B glycogen synthase kinase 3 beta -4.55

WNT4 wingless-type MMTV integration site family, member 4 -4.36

FZD7 frizzled homolog 7 (Drosophila) 3.81

WNT5A wingless-type MMTV integration site family, member 5A 3.59

NFATC1 nuclear factor of activated T-cells, cytoplasmic,

calcineurin-dependent 1 3.57

WNT7B wingless-type MMTV integration site family, member 7B -3.09

FZD6 frizzled homolog 6 (Drosophila) -2.93

DVL2 dishevelled, dsh homolog 2 (Drosophila) 2.54

FZD1 frizzled homolog 1 (Drosophila) 2.26

DKK3 dickkopf homolog 3 (Xenopus laevis) 2.24

DVl1 dishevelled, dsh homolog 1 (Drosophila) -2.47

WNT10A wingless-type MMTV integration site family, member 10A -2.86

WNT11 wingless-type MMTV integration site family, member 11 2.36

LRP5 low density lipoprotein receptor-related protein 5 -1.58

**Notch signaling**

GCN5L2 GCN5 general control of amino-acid synthesis 5-like 2 (yeast) 49.89

Hey1 hairy/enhancer-of-split related with YRPW motif 1 30.10

Hey2 hairy/enhancer-of-split related with YRPW motif 2 24.68

DLL1 delta-like 1 (Drosophila) 10.64

HeyL hairy/enhancer-of-split related with YRPW motif-like 7.08

Hes7 hairy and enhancer of split 7 (Drosophila) -4.32

Hes2 hairy and enhancer of split 2 (Drosophila) -3.84

JAG1 jagged 1 (Alagille syndrome) -3.03

DTX3 deltex 3 homolog (Drosophila) 3.02

Hes6 hairy and enhancer of split 6 (Drosophila) 2.79

Hes5 hairy and enhancer of split 5 (Drosophila) 2.75

DLL3 delta-like 3 (Drosophila) 2.63

MAML mastermind-like 1 (Drosophila) 2.51

NUMB numb homolog (Drosophila) -2.44

Notch3 Notch homolog 3 (Drosophila) 2.37

Hes4 hairy and enhancer of split 4 (Drosophila) 2.34

PSEN1 presenilin 1 (Alzheimer disease 3) -2.16

Notch1 Notch homolog 1, translocation-associated (Drosophila) 1.94

JAG2 jagged 2 1.71

**TGF-beta signaling pathway**

THBS1 thrombospondin 1 -88.29

ID2 inhibitor of DNA binding 2, dominant negative

helix-loop-helix protein 22.80

TGFB2 transforming growth factor, beta 2 -17.06

ID3 inhibitor of DNA binding 3, dominant negative

helix-loop-helix protein 15.48

BMP7 bone morphogenetic protein 7 (osteogenic protein 1) 14.82

BMP2 bone morphogenetic protein 2 12.74

INHBB inhibin, beta B -10.72

PITX2 paired-like homeodomain 2 -9.31

TGFBR2 transforming growth factor, beta receptor II (70/80kDa) -8.93

SMAD6 SMAD family member 6 7.45

BMP6 bone morphogenetic protein 6 5.19

SMAD7 SMAD family member 7 3.41

SMAD3 SMAD family member 3 3.19

SMAD9 SMAD family member 9 2.37

SMAD4 SMAD family member 4 2.30

SMAD5 SMAD family member 5 2.24
