# Supplementary material for: Epithelial to Mesenchymal Transition Is Mechanistically Linked with Stem Cell Signatures in Prostate Cancer Cells
Source: PLoS One. 2010 Aug 27;5(8):e12445. doi: 10.1371/journal.pone.0012445 (PMC2929211; doi:10.1371/journal.pone.0012445)
Supplement: Table S3 — The levels of miR-200 and let-7 family in PC3 Neo and PC3 PDGF-D cells. (0.02 MB DOC) [file pone.0012445.s008.doc]

Table S3: The levels of miR-200 and let-7 family in PC3 Neo and PC3 PDGF-D cells.

Name Mean (PC3 Neo) Mean (PC3 PDGF-D) Log2*

hsa-miR-200a 193 4 -5.7

hsa-miR-200b 1474 4 -8.56

hsa-miR-200c 20 9 -1.14

hsa-miR-429 229 5 -5.48

hsa-let-7a 15277 4180 -1.87

hsa-let-7b 2212 327 -2.76

hsa-let-7c 5657 1709 -1.73

hsa-let-7d 8037 1628 -2.3

hsa-let-7e 3305 1200 -1.46

hsa-let-7f 9851 2534 -1.96

hsa-let-7g 3084 1195 -1.37

hsa-let-7i 5110 369 -3.79

* PC3 PDGF-D/PC3 Neo
