# Supplementary material for: Epithelial to Mesenchymal Transition Is Mechanistically Linked with Stem Cell Signatures in Prostate Cancer Cells
Source: PLoS One. 2010 Aug 27;5(8):e12445. doi: 10.1371/journal.pone.0012445 (PMC2929211; doi:10.1371/journal.pone.0012445)
Supplement: Table S4 — Primer sequences used in real time PCR. (0.04 MB DOC) [file pone.0012445.s009.doc]

Table S4: Primer sequences used in real time PCR

| Primer Name | Sequence |
| --- | --- |
| GAPDH forward  GAPDH reverse  Oct4 forward  Oct4 reverse  Nanog forward  Nanog reverse  Sall4 forward  Sall4 reverse  Zic2 forward  Zic2 reverse  Sox2 forward  Sox2 reverse  Sall2 forward  Sall2 reverse  Sox3 forward  Sox3 reverse  Zic3 forward  Zic3 reverse  Sox17 forward  Sox17 reverse  Stat3 forward  Stat3 reverse  Sox11 forward  Sox11 reverse  Lin28B forward  Lin28B reverse  EED forward  EED reverse  Ezh2 forward  Ezh2 reverse  Suz12 forward  Suz12 reverse  Hes1 forward  Hes1 reverse  Notch2 forward  Notch2 reverse Notch3 forward  Notch3 reverse  Hes6 forward  Hes6 reverse  Hes4 forward  Hes4 reverse  Notch1 forward  Notch1 reverse  Hey1 forward  Hey1 reverse  Hey2 forward  Hey2 reverse  HeyL forward  HeyL reverse  Hes5 forward  Hes5 reverse | 5'-ACCCAGAAGACTGTGGATGG-3'  5'-CAGTGAGCTTCCCGTTCAG-3'  5'-GTCCGAGTGTGGTTCTGTA -3'  5'-CTCAGTTTGAATGCATGGGA-3'  5'-caaaggcaaacaacccactt-3'  5'- tctgctggaggctgaggtat -3'  5'- tgatcccaacgaatgtctca-3'  5'- cccaaggtgtgtcttcaggt-3'  5'- aatcccaagaagagctgcaa-3'  5'- cactcctcccagaagcagac-3'  5'- atgggttcggtggtcaagt-3'  5'- gctctggtagtgctgggaca-3'  5'- atcaagcctgtccaaaccag -3'  5'-cctccagcagagaaaggatg-3'  5'- agaccaggaccgtgtgaaac-3'  5'- gtcgatgaatggtcgcttct-3'  5'- catacctcggacaagcccta-3'  5'- tatagcgggtggagtggaag-3'  5- tttcatggtgtgggctaagg-3'  5'-cccagcatcttgctcaactc-3'  5'- ACATTCTGGGCACAAACACA -3'  5'- CAGTCACAATCAGGGAAGCA -3'  5'- tgatgttcgacctgagcttg -3'  5'- ctcgctgaacgaatccaaat -3'  5'- gcaaaggtggtggagaagag -3'  5'- ggcttccctctcggtttatc -3'  5'- tgacgagaacagcaatccag -3'  5'- ggtgtatcagggcgttcagt -3'  5'- aggagtttgctgctgctctc -3'  5'- gggcctgctactgttattgg -3'  5'- agtagccatgcaggaaatgg -3'  5'- gtttggcaataggagccgta -3'  5'- aacacgacaccggataaacc -3'  5'- ccgcgagctatctttcttca -3'  5'- gaagggagcacctgtgagag -3'  5'- gcggcagttgtaagtgttga -3'  5'- gtcgtggctacactggacct -3'  5'- aatgtccacctcgcaatagg -3'  5'- ccctgaggctgaactgagtc -3'  5'- aatttgggctgtggtcagg -3'  5'- gagcgcgtattaacgagagc -3'  5'- ctcacggtcatctccaggat -3'  5'- gtaccgaggatgtggacgag -3'  5'- acacacacgcagttgtagcc -3'  5'- cgcccttgctatggactatc -3'  5'- ttgttgagatgcgaaaccag -3'  5'- gcataggattccgagagtgc -3'  5'- cgcaagtgctgagatgagac -3'  5'- atagagaaacggcgtcgaga -3'  5'- tttcaagtgatccaccgtca -3'  5'- acatcctggagatggctgtc -3'  5'- tagtcctggtgcaggctctt -3' |
